# Segmenting subregions of the human hippocampus on structural magnetic resonance image scans: An illustrated tutorial

**DOI:** 10.1177/2398212817701448

**Published:** 2017-04-06

**Authors:** Marshall A. Dalton, Peter Zeidman, Daniel N. Barry, Elaine Williams, Eleanor A. Maguire

**Affiliations:** Wellcome Trust Centre for Neuroimaging, Institute of Neurology, University College London, London, UK

**Keywords:** Hippocampus, subfields, magnetic resonance imaging, segmentation, high-resolution, histology

## Abstract

**Background:**

The hippocampus plays a central role in cognition, and understanding the specific contributions of its subregions will likely be key to explaining its wide-ranging functions. However, delineating substructures within the human hippocampus in vivo from magnetic resonance image scans is fraught with difficulties. To our knowledge, the extant literature contains only brief descriptions of segmentation procedures used to delineate hippocampal subregions in magnetic resonance imaging/functional magnetic resonance imaging studies.

**Methods:**

Consequently, here we provide a clear, step-by-step and fully illustrated guide to segmenting hippocampal subregions along the entire length of the human hippocampus on 3T magnetic resonance images.

**Results:**

We give a detailed description of how to segment the hippocampus into the following six subregions: dentate gyrus/Cornu Ammonis 4, CA3/2, CA1, subiculum, pre/parasubiculum and the uncus. Importantly, this in-depth protocol incorporates the most recent cyto- and chemo-architectural evidence and includes a series of comprehensive figures which compare slices of histologically stained tissue with equivalent 3T images.

**Conclusion:**

As hippocampal subregion segmentation is an evolving field of research, we do not suggest this protocol is definitive or final. Rather, we present a fully explained and expedient method of manual segmentation which remains faithful to our current understanding of human hippocampal neuroanatomy. We hope that this ‘tutorial’-style guide, which can be followed by experts and non-experts alike, will be a practical resource for clinical and research scientists with an interest in the human hippocampus.

## Introduction

The human hippocampus is implicated in a wide range of cognitive processes including episodic memory, future thinking, navigation and aspects of perception (Maguire and Mullally, 2013; Schacter et al., 2012; Zeidman and Maguire, 2016). Its involvement in a broad spectrum of functions is not surprising given its widespread connectivity with other brain regions (Braak et al., 1996) and the complexity of its internal structure which is not homogenous but contains subregions including the dentate gyrus (DG), Cornu Ammonis (CA) 4-1 and the subicular cortices. Importantly, a growing body of evidence suggests that these subregions have unique patterns of connectivity and may be differentially implicated in disparate aspects of cognition (Coras et al., 2014; Zeidman et al., 2015), although as yet we lack a detailed understanding of their precise contributions.

Limitations in magnetic resonance image (MRI) resolution have necessarily restricted the ability of researchers to investigate human hippocampal subregions in vivo. Typically, questions relating to hippocampal function are addressed by coarsely segmenting anterior and posterior portions of the hippocampus (Collin et al., 2015; Poppenk et al., 2013; Satpute et al., 2012; Strange et al., 2014). More recent advances in high-resolution image acquisition have presented opportunities to examine the hippocampus in greater detail and create subregion-specific masks which facilitate more comprehensive investigations (Wisse et al., 2012; Zeidman et al., 2015). While high-resolution images acquired on 7-T scanners provide excellent neuroanatomical detail (e.g. Iglesias et al., 2015), such scanners are not yet widely available. However, acquisition of high-resolution images of sufficient quality to segment hippocampal subregions is now possible on more readily available 3T scanners.

Methods for accurately delineating hippocampal subregions on high-resolution images are still in development. Decisions made in relation to the location of borders between subregions vary greatly among researchers (Yushkevich et al., 2015), and there is currently no widely agreed protocol for manually segmenting subregions along the entire length of the hippocampus. Furthermore, there is considerable inter-subject variability in the morphology of the hippocampus – for instance, people generally have between three and five digitations or folds in the dorsal portion of the anterior hippocampus (Ding and Van Hoesen, 2015). Indeed, variability in the appearance of both the anterior and posterior hippocampus on MRI scans can make subfield delineation challenging. We give further consideration to this important issue in the ‘Discussion’ section. These factors limit the accuracy of software purporting to perform automated hippocampal subregion segmentation for the whole hippocampus. One possible explanation for the variability across manual segmentation schemes is that hippocampal subregion borders cannot always be seen on MRI scans, even at 7T. Rather, their location must be inferred by applying neuroanatomical knowledge gained from histological investigations of the hippocampus to MRIs. While some hippocampal landmarks can be observed on MRIs, tissue intensity fluctuations inherent to these scans result in regions of ambiguity, making identification of subregions difficult. This is especially true in the anterior and posterior portions of the hippocampus. Arguably, therefore, one of the most important tools for hippocampal subregion segmentation on MRI scans is an understanding of hippocampal neuroanatomy at the microscopic level. Developing a mental scaffold at the microscopic level is crucial for making sense of the ambiguities inherent to MRIs.

While a number of articles describe the methods that they used to delineate hippocampal subregions on MRIs acquired on 3T (Bonnici et al., 2012; Kulaga-Yoskovitz et al., 2015; La Joie et al., 2013; Olsen et al., 2013; Palombo et al., 2013; Van Leemput et al., 2009; Winterburn et al., 2013, 2015; Wood et al., 2015; Zeidman et al., 2015), 4T (Das et al., 2012; Mueller et al., 2007), 4.7T (Malykhin et al., 2010), 7T (Berron et al., 2016; Iglesias et al., 2015; Wisse et al., 2012) and 9.4T (Adler et al., 2012, 2014; Yushkevich et al., 2009) scanners, most provide a relatively brief description and justification for their border delineations. Typically, researchers cite a collection of core standard texts which are used for guidance. These commonly include, but are not limited to, Duvernoy et al. (2013), Amaral and Lavanex (2007) and Mai et al. (2008). While these texts provide thorough foundational insights for subregion segmentation, our understanding of hippocampal subregions continues to evolve. Recent cyto- and chemo-architectural investigations offer additional lines of evidence for border delineations. Of particular note, Ding and Van Hoesen (2015) provide what is arguably the most detailed anatomical description of the human anterior hippocampus to date. Studies such as these extend the knowledge available in the core texts and should be incorporated into our models of subregion segmentation.

**Figure 1.**
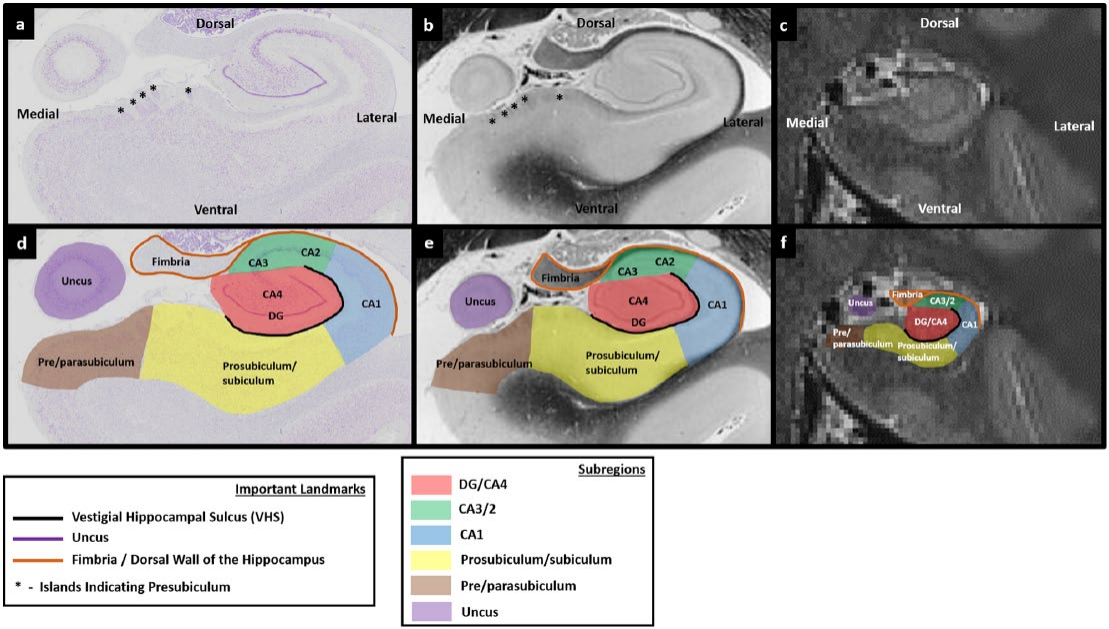
Subregions of the human hippocampus. (a) A section of post-mortem human hippocampus stained with cresyl violet to visualise cell bodies and (b) an equivalent slice of post-mortem human hippocampus stained with haematoxylin (Weigert) to visualise white matter. Both sections are from ‘The Human Brain’ website http://www.thehumanbrain.info/brain/sections.php. (c) A T2-weighted structural MRI of the human hippocampus. This section is approximately equivalent to the slice represented in ‘a’ and ‘b’. (d) The same section is presented as in ‘a’ but now overlaid with hippocampal subregion masks. (e) The same section is presented as in ‘b’ but now overlaid with hippocampal subregion masks. (f) The same section is presented as in ‘c’ but now overlaid with hippocampal subregion masks.

Surprisingly, to our knowledge, there is nothing in the literature that lays out in a clear, step-by-step and fully illustrated manner, a method for segmenting hippocampal subregions along the entire length of the human hippocampus on 3T MRIs. While one article provides a description of a segmentation protocol (Winterburn et al., 2015), it does not incorporate the latest neuroanatomical data relating to the anterior hippocampus, nor does it provide a detailed step-by-step guide which is immediately useful to those with and, in particular, those without prior experience of hippocampal anatomy. The aim of this article is to provide such a guide. This in-depth protocol incorporates recent cyto- and chemo-architectural evidence, especially in relation to the anterior portion of the hippocampus. We give a detailed description of how to segment the hippocampus into the following six subregions: DG/CA4, CA3/2, CA1, prosubiculum/subiculum, pre/parasubiculum and the uncus (Figure 1) – the latter two being highlighted as functionally differentiable in recent human functional MRI (fMRI) studies (Zeidman et al., 2015; Zeidman and Maguire, 2016). Importantly, to aid the reader, we also include a series of comprehensive figures which compare slices of histologically stained tissue with equivalent 3T images.

We are not proposing that this protocol is definitive or final. Rather, this represents one fully explained and expedient method of manual segmentation which remains faithful to our current understanding of human hippocampal biology. Moreover, this is not intended as an exhaustive review of hippocampal neuroanatomy, an atlas or automated software tool. Instead, we describe those aspects of anatomy which can be used as markers to identify the likely location of borders between subregions of the hippocampus from data acquired using a high-resolution T2-weighted structural MRI sequence that can be implemented on most 3T scanners.

As noted by Iglesias et al. (2015), two of the main obstacles to a research group’s ability to reliably engage in manual hippocampal subregion delineation are a lack of neuroanatomical expertise and the time-consuming nature of the work. We hope that clinical and research scientists with an interest in the hippocampus will find our method useful and expedient until a broader consensus is reached within the field. Similarly, beginners may find this tutorial helpful in developing expertise. We used the 3T high-resolution T2-weighed structural MRI sequence that we find to be the most effective, but this protocol can offer guidance even when other sequences are employed.

In summary, manual segmentation is the gold standard when analysing structural MRI scans of human hippocampal subregions. As yet, a field-wide consensus is lacking on precisely how to accomplish this. In the meantime, there is an urgent need and desire to conduct research on human hippocampal subregions. To facilitate this, we describe here a comprehensive and practical guide that explains how to perform hippocampal segmentation, the reasoning behind our decisions and consideration of the issues and challenges involved.

## Materials and methods

### Image acquisition

Data were acquired on a 3T whole-body MRI scanner (Magnetom TIM Trio; Siemens Healthcare, Erlangen, Germany) operated with a radiofrequency (RF) transmit body coil and 32-channel head RF receive coil. Imaging was limited to a partial volume focused on the temporal lobes. These structural images were collected using a single-slab three-dimensional (3D) T2-weighted turbo spin echo sequence with variable flip angles (SPACE) (Mugler et al., 2000) in combination with parallel imaging, to simultaneously achieve a high image resolution of ~500 µm, high sampling efficiency and short scan time while maintaining a sufficient signal-to-noise ratio (SNR). After excitation of a single axial slab, the image was read out with the following parameters: resolution = 0.52 × 0.52 × 0.5 mm, matrix = 384 × 328, partitions = 104, partition thickness = 0.5 mm, partition oversampling = 15.4%, field of view = 200 × 171 mm^2^, TE = 353 ms, TR = 3200 ms, GRAPPA × 2 in phase-encoding (PE) direction, bandwidth = 434 Hz/pixel, echo spacing = 4.98 ms, turbo factor in PE direction = 177, echo train duration = 881 and averages = 1.9. For reduction of signal bias due to, for example, spatial variation in coil sensitivity profiles, the images were normalised using a prescan, and a weak intensity filter was applied as implemented by the scanner’s manufacturer. To improve the SNR of the anatomical image, three scans were acquired for the participant (who was a healthy 24-year-old female). It took 12 min to obtain each scan with a total scanning time of 36 min. The images were co-registered and denoised following the Rician noise estimation (Coupe et al., 2010). The denoised images were averaged and smoothed with a full-width at half maximum kernel of 2 × 2 × 2 mm. Images were segmented using ITK-SNAP (Yushkevich et al., 2006).

### Histological sections

High-resolution images of histologically stained coronal sections of medial temporal lobe (MTL) were acquired using the virtual microscopy tool on ‘The Human Brain’ website http://www.thehumanbrain.info/brain/sections.php. This freely available tool provides slices that have been stained with cresyl violet to visualise cell bodies and haematoxylin (Weigert) to visualise white matter. Note that the histological sections are not from the MRI participant. Consequently, although we have attempted to provide closely equivalent post-mortem and MRI slices in each figure, occasional inconsistencies may exist between the two.

### Segmentation protocol

**Figure 2.**
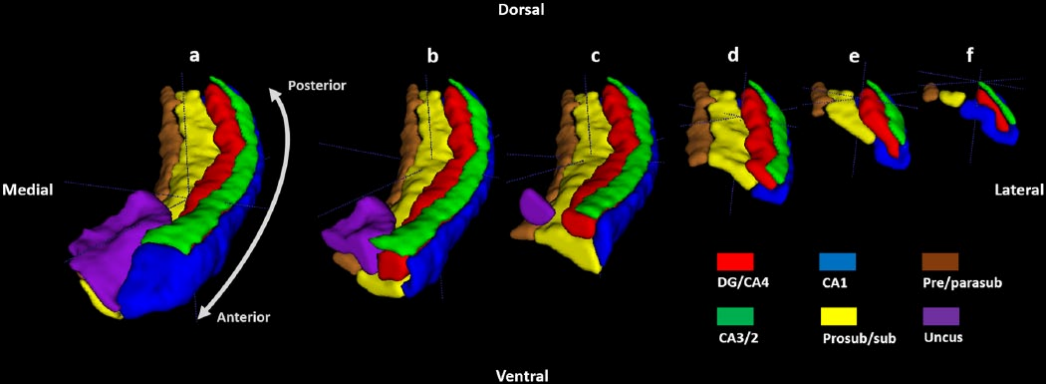
A 3D model of hippocampal subregion masks. Presented in their entirety (a) and with anterior slices progressively removed in an anterior-to-posterior direction to reveal internal subregion structure (b)–(f).

**Figures 3.**
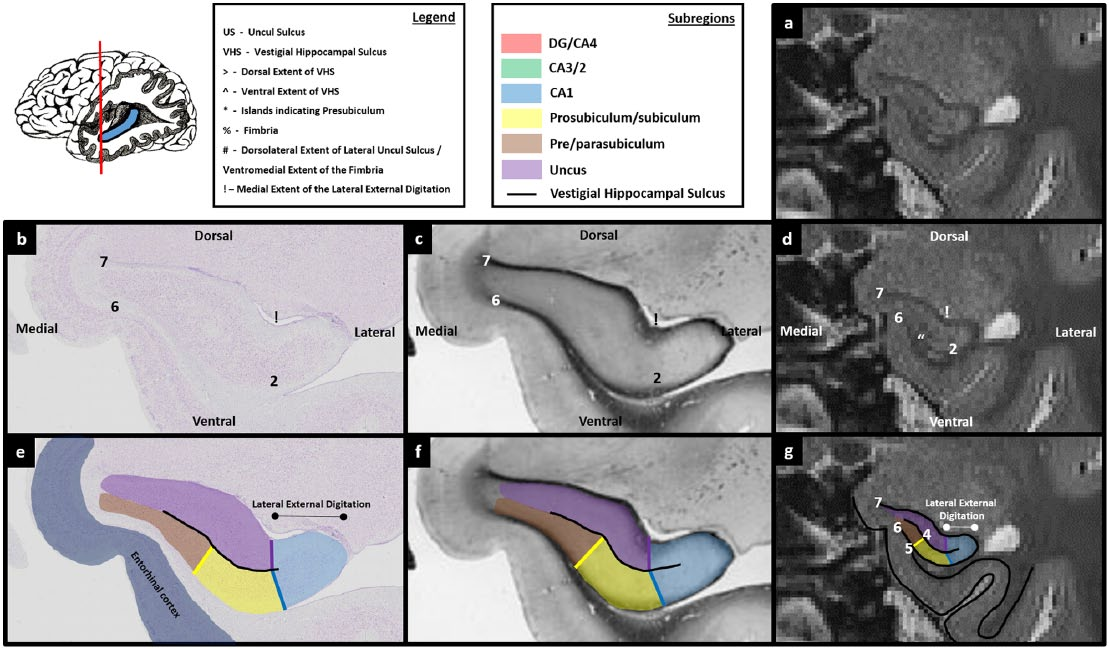
Comparison of post-mortem tissue and T2-weighted images. Each of these figures contains the following: (a) a T2-weighted structural MRI of the human hippocampus and (b) a section of post-mortem human hippocampus stained with cresyl violet to visualise cell bodies. This section is approximately equivalent to the T2-weighted MRI presented in ‘a’. Salient landmarks referred to in the text are marked on the image. (c) Equivalent slice of post-mortem human hippocampus stained with haematoxylin (Weigert) to visualise white matter. Salient landmarks referred to in the text are marked on the image; ‘b’ and ‘c’ are from ‘The Human Brain’ website http://www.thehumanbrain.info/brain/sections.php. (d) This is the same T2-weighted MRI as presented in ‘a’ but now with salient landmarks referred to in the text marked on the image. (e) This is the same section as presented in ‘b’ but now overlaid with hippocampal subregion masks. (f) This is the same section as presented in ‘c’ but now overlaid with hippocampal subregion masks. (g) This is the same section as presented in ‘a’ and ‘d’ but now overlaid with hippocampal subfield masks. Note that this legend also pertains for Figures 4-15.

**Figure 4:**
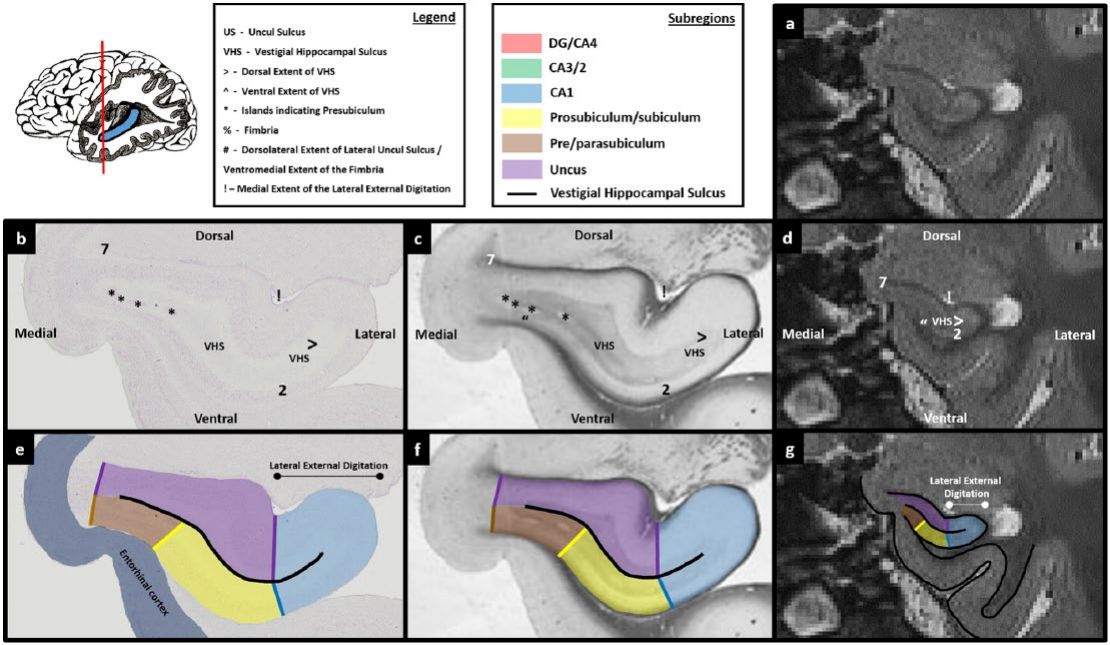


**Figure 5:**
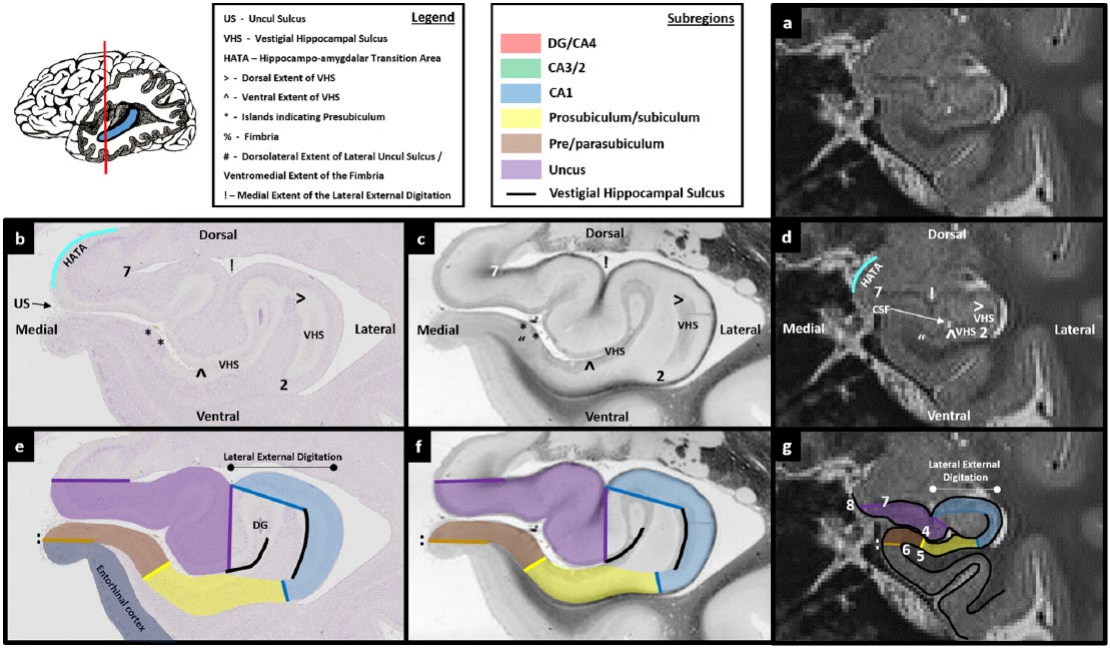


**Figure 6:**
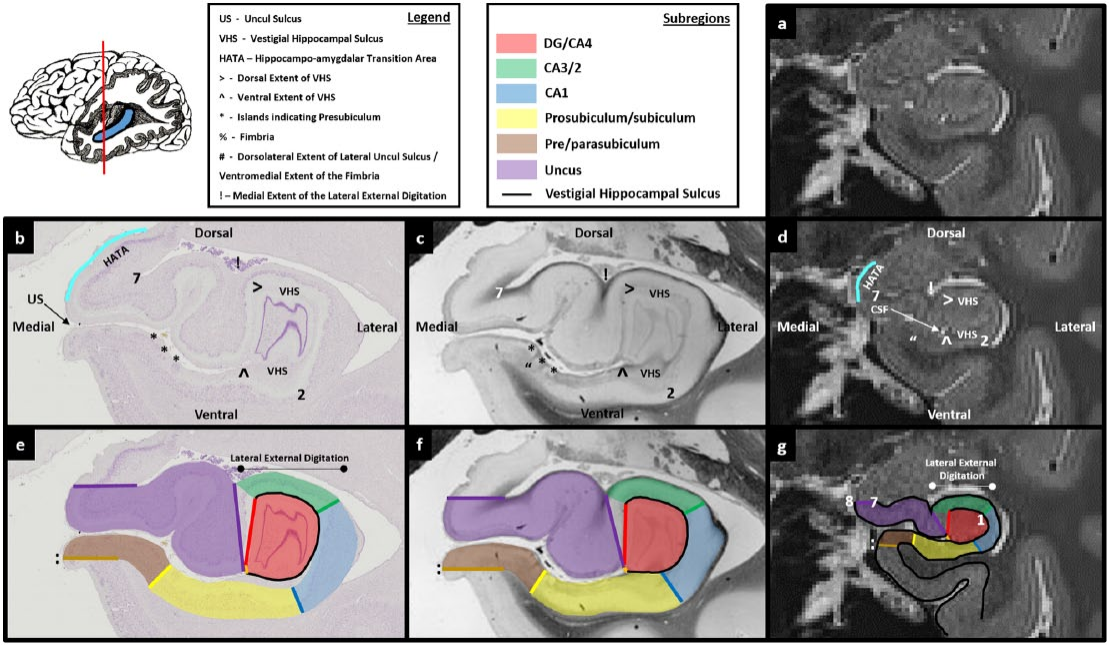


**Figure 7:**
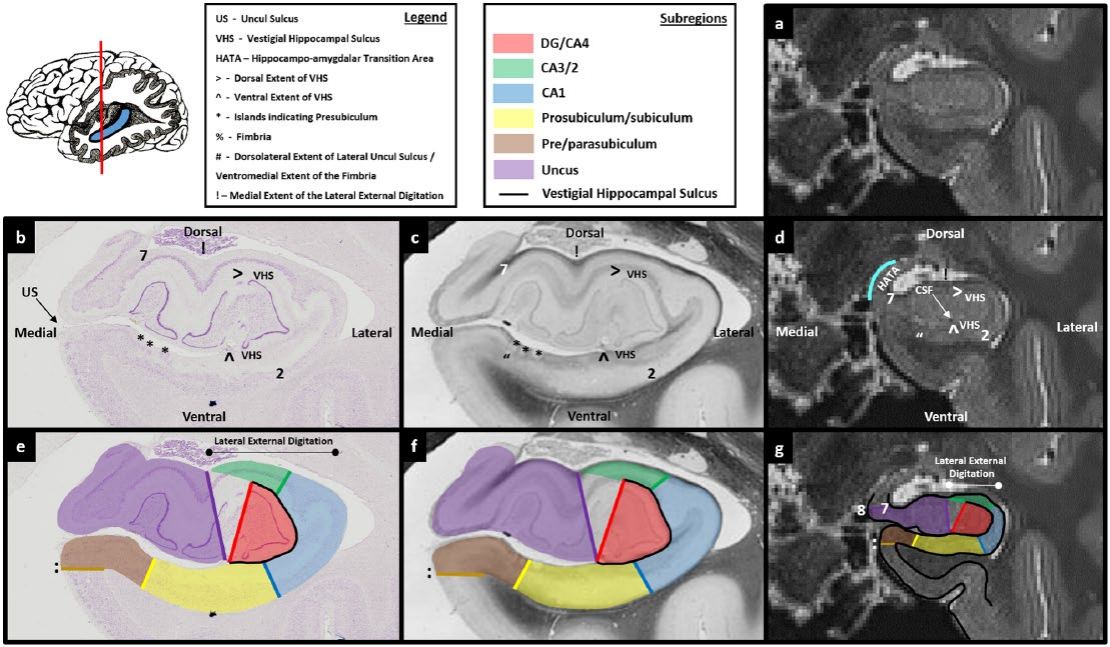


**Figure 8:**
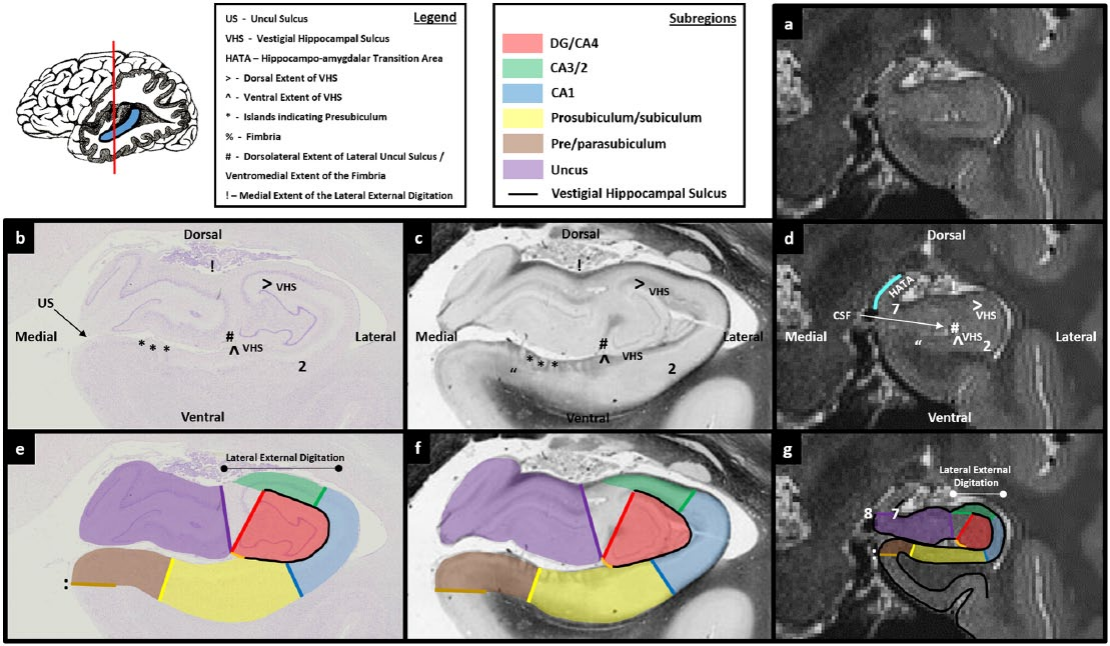


**Figure 9:**
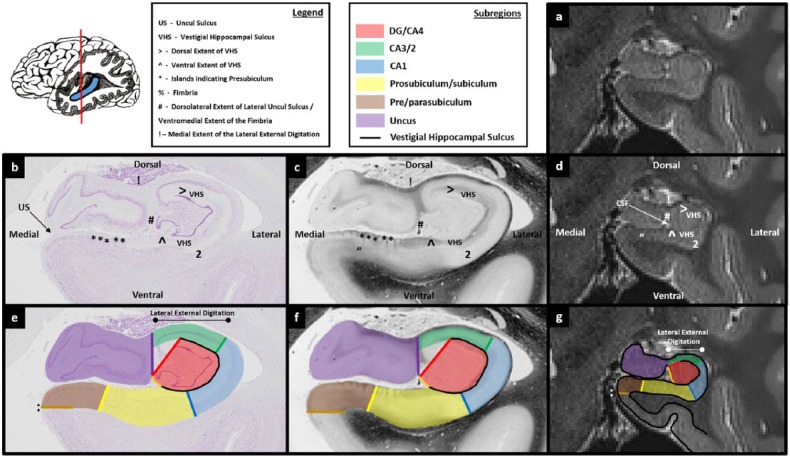


**Figure 10:**
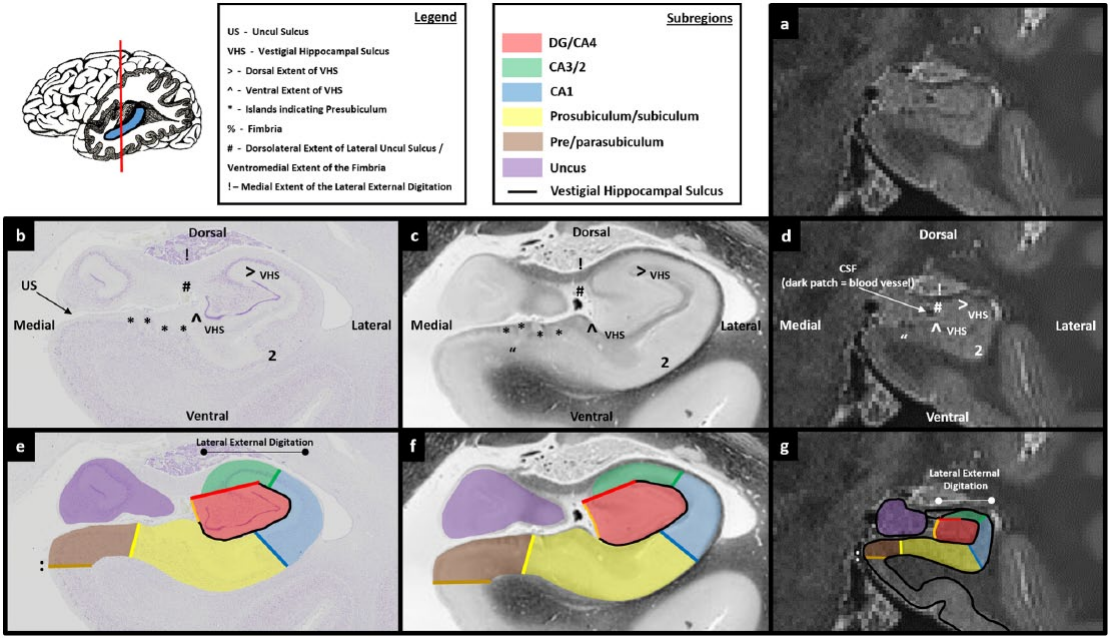


**Figure 11:**
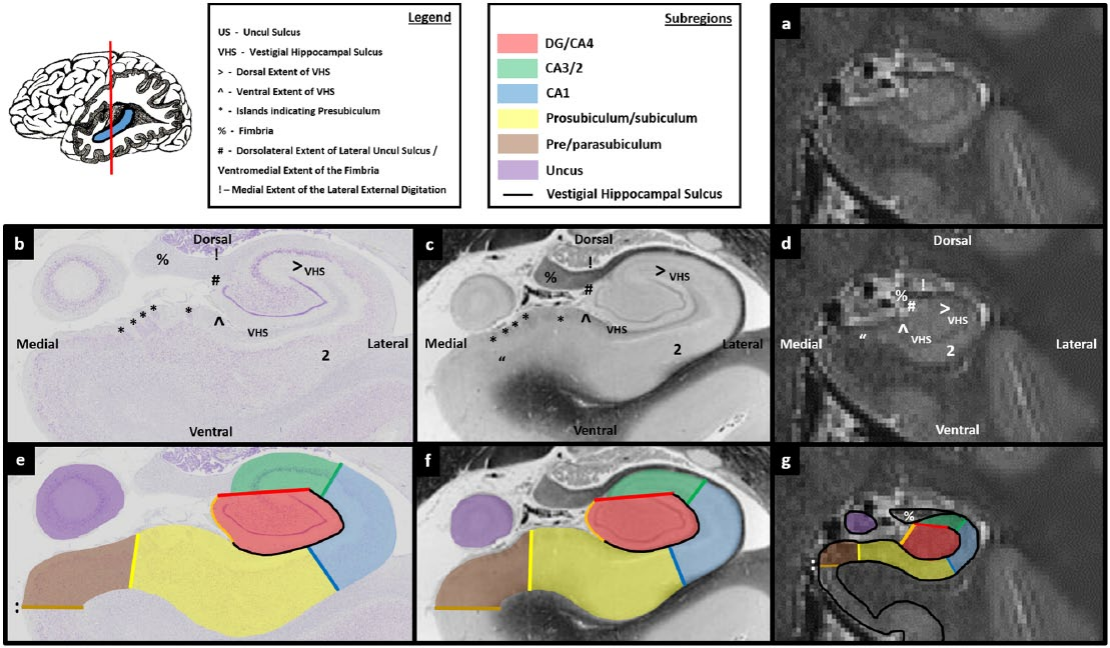


**Figure 12:**
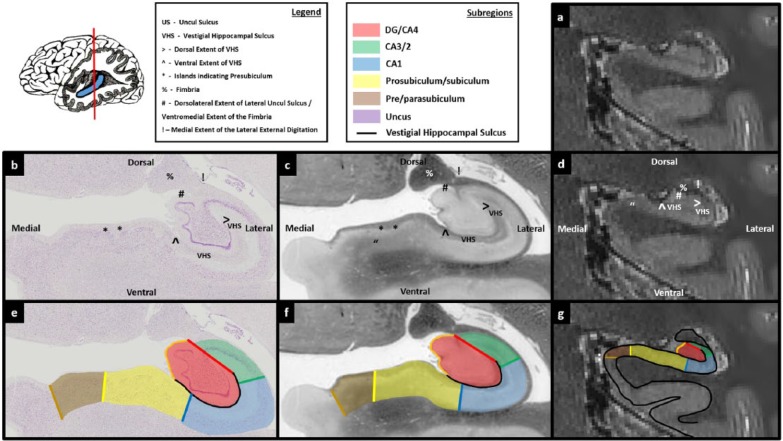


**Figure 13:**
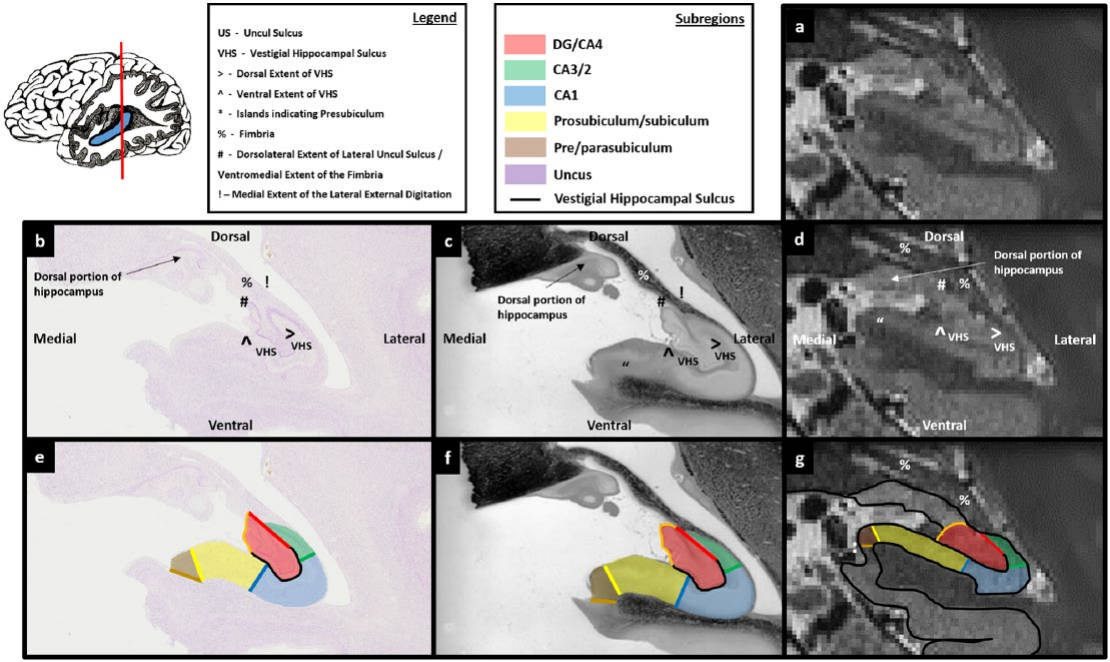


**Figure 14:**
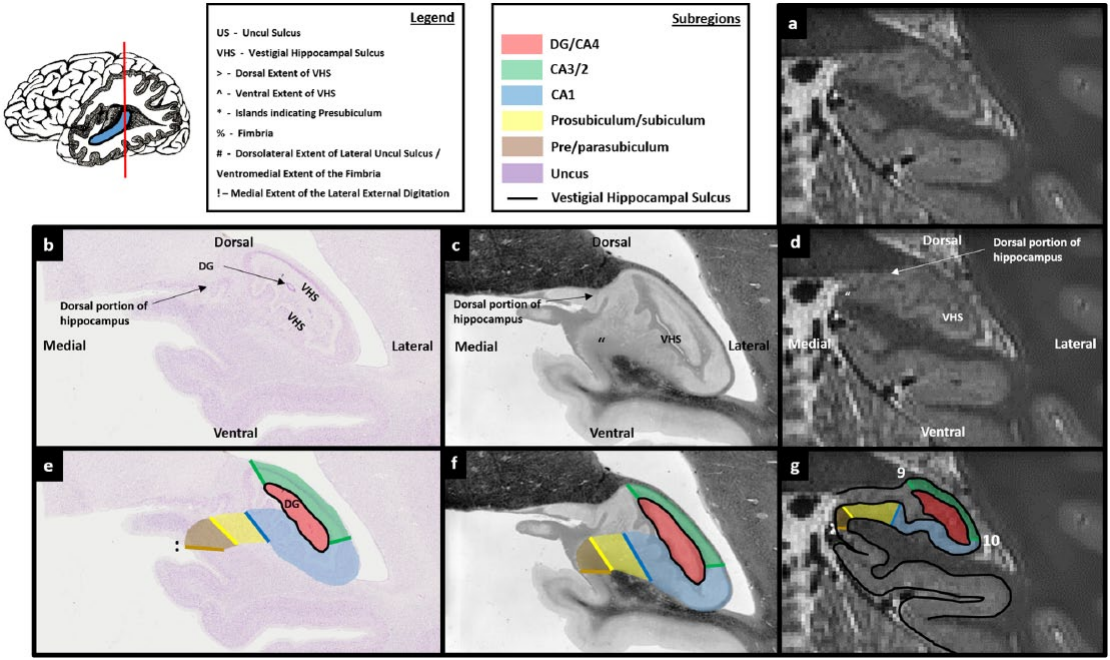


**Figure 15:**
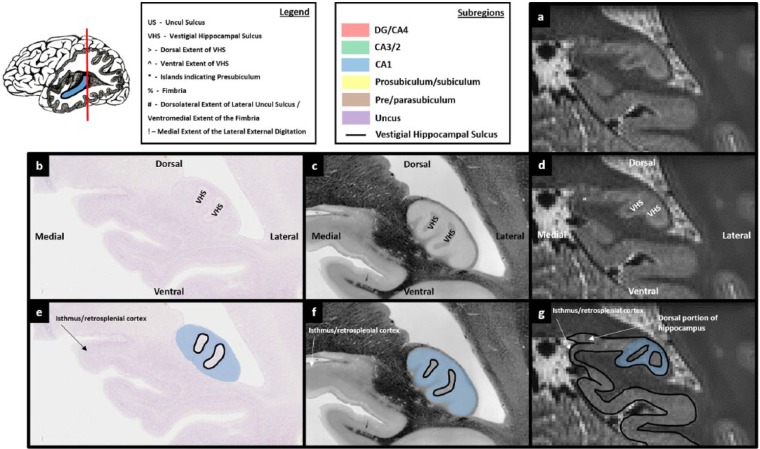


**Figure 16.**
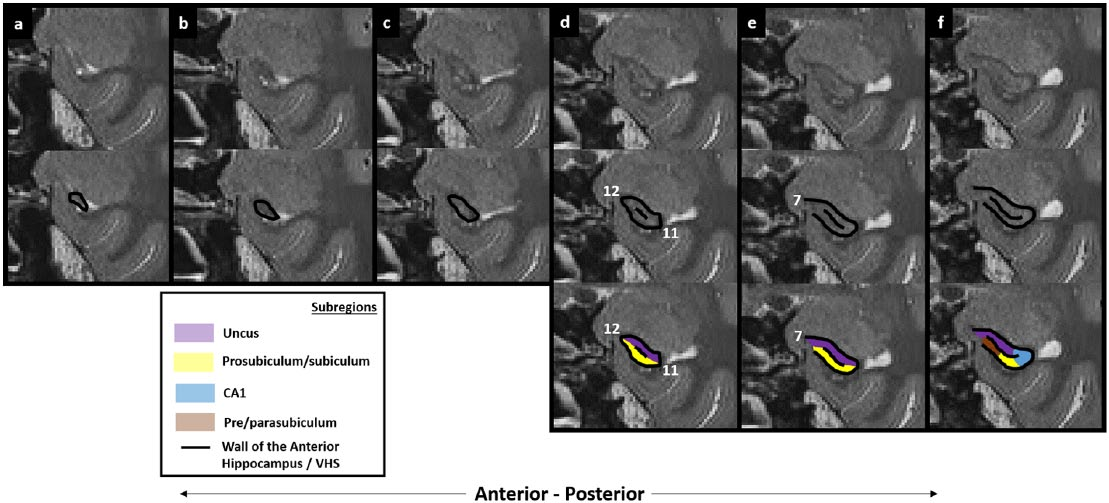
Subregion delineation in the anterior-most portions of the hippocampus.

**Figure 17.**
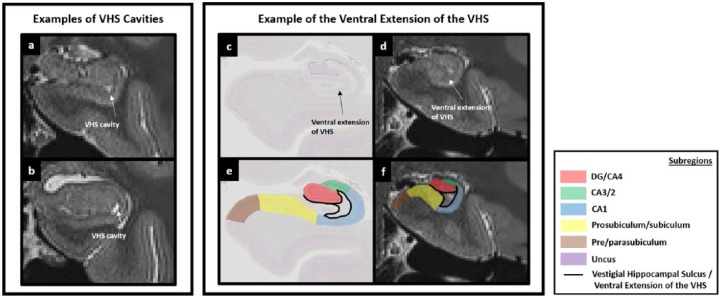
Potential issues relating to the vestigial hippocampal sulcus.

**Figures 18.**
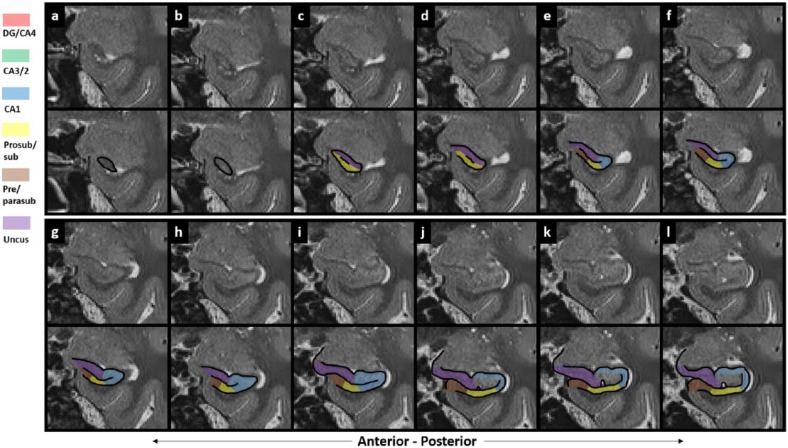
Subregion delineations across the longitudinal axis of the hippocampus. These figures show a slice-by-slice sequence of images spanning the longitudinal axis of the hippocampus in its entirety, overlaid with the subregion delineations. Within each figure, ‘a’ represents the anterior-most slice, with each subsequent panel (‘b’, ‘c’, ‘d’, etc.) representing a contiguous slice in the posterior direction. For each figure, the slice following the final panel ‘l’ is continued in ‘a’ of the following figure. Note that the same legend pertains for Figures 19-24.

**Figure 19:**
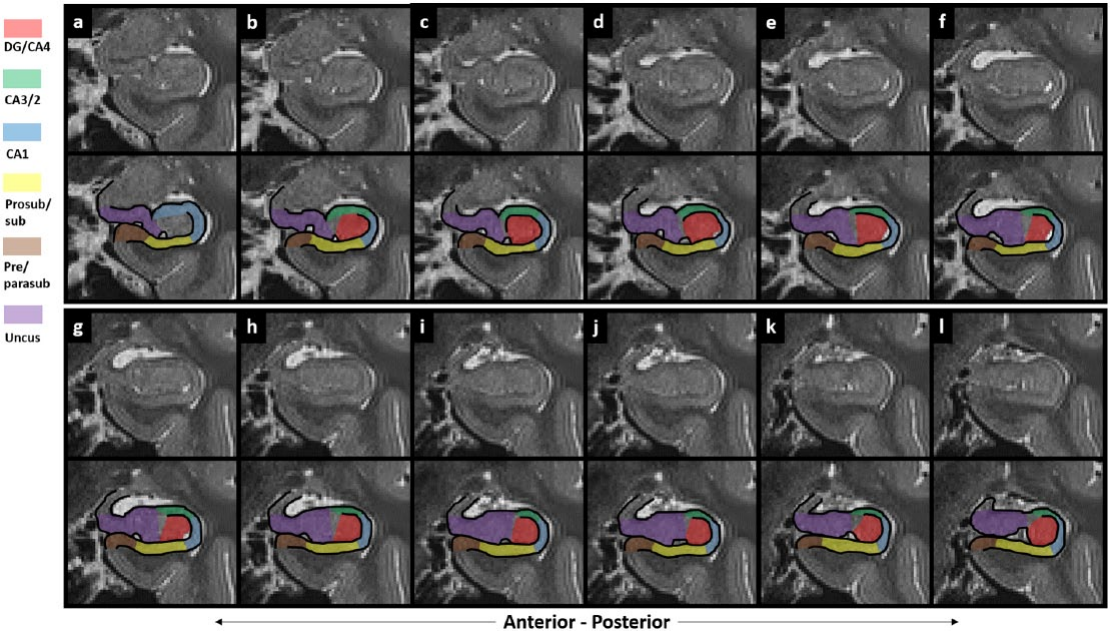


**Figure 20:**
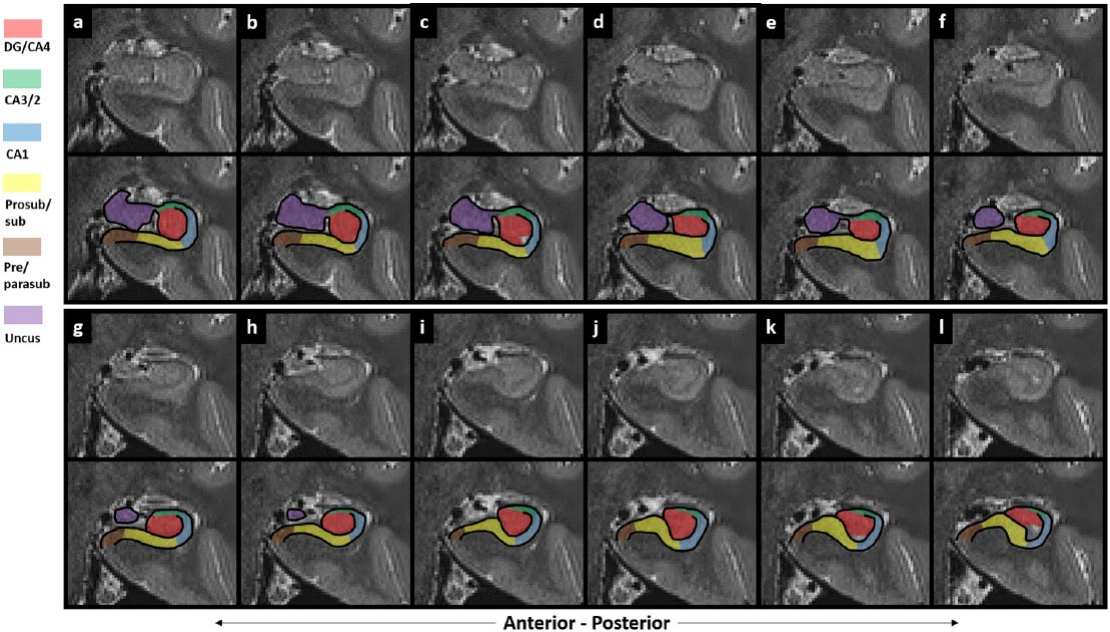


**Figure 21:**
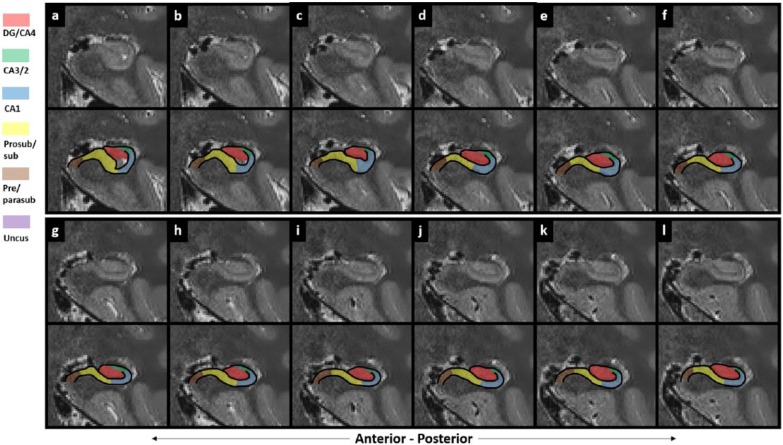


In this protocol, which is in the coronal plane, we utilise a mixture of geometric rules and to a lesser extent intensity contrasts. For the most part, we attempt to use anatomical landmarks to infer the approximate location of each boundary but it is important to acknowledge that ‘top–down’ processing on the part of the experimenter is necessary when making decisions about MRIs. Before attempting to find any landmark described here, it is good practice to first develop a clear understanding of where they lie on coronal sections of histologically stained tissue. A thorough knowledge of and reference to the underlying neuroanatomy is crucial and can facilitate the decision-making process on more difficult slices. We recommend keeping the figures readily at hand when segmenting. Note that a companion document containing just the figures is available here (http://www.fil.ion.ucl.ac.uk/Maguire/Dalton_Maguire_BNA_Figures_1-29.pdf). They are intended to be used as a guide and troubleshooting tool. We adopt an instructive tone throughout the protocol description. This is not meant to imply that this protocol is definitive or final, but is merely for practical purposes.

## Results

There are six parts to the protocol (Figure 1), corresponding to DG/CA4, CA3/2, CA1, prosubiculum/subiculum, pre/parasubiculum and the uncus. To help orient the experimenter, a 3D rendered example of the final product of this segmentation protocol is shown in Figure 2, where the characteristic elongated structure of the hippocampus extending in an anterior–posterior direction can be observed. Within each part of the protocol, there are three (DG/CA4, CA3/2, CA1) or two (subiculum, pre/parasubiculum, uncus) sub-sections describing salient divisions. Each sub-section is prefaced by consideration of the relevant neuroanatomy as revealed by histology and its applicability to T2-weighted MRIs, which is then proceeded by descriptions of the key steps necessary to execute the segmentation. In each of the coronal slices which accompany the text, the right hemisphere of the brain is shown. As such, the medial–lateral axis goes from left to right in each image (see labels in Figure 1(a)), meaning that the left of each image is closest to the brain’s midline. We are not aware of any major structural differences between left and right hippocampus, so instructions in this guide may be ‘flipped’ to segment the left hippocampus.

## Part 1: the DG/CA4 mask

We recommend creating the DG/CA4 mask first, as subsequent subregion delineations can be made in relation to this mask. The DG itself cannot be seen on T2-weighted images at this resolution. We must, therefore, rely on knowledge of the underlying neuroanatomy for clues with which to identify its position. This is especially important when deciding which slices are likely to contain the anterior- and posterior-most extents of the DG. Of note, although CA4 is also commonly referred to as the hilus region of the hippocampus, we use the term CA4 for this region throughout the protocol.

### First slice of the DG/CA4 mask

#### Histology

The first useful marker relates to the lateral portion of the anterior hippocampus. At its most anterior extent, the hippocampus appears as a thin ribbon of tissue in the MTL (Figure 3(b) and (c)). Starting at the anterior-most tip of the hippocampus and moving in a posterior direction, the lateral extent of the hippocampus begins to fatten and becomes rounder (Figures 3–6(b) and (c) sequentially – note the gradual fattening of the lateral external digitation of the hippocampus). Soon after this fattening occurs, the DG emerges and fills the centre of the lateral portion of the hippocampus (Figure 6(b)). The emergence of the DG is preceded by this characteristic fattening of the lateral portion of the anterior hippocampus, which can therefore be used to help identify the anterior-most slice in which the DG is likely to be present on T2-weighted images (see Step 1).

The second useful marker for the DG/CA4 border relates to the vestigial hippocampal sulcus (VHS). This is also commonly referred to as the hippocampal fissure and is bordered by the stratum radiatum and stratum lacunosum-moleculare (sometimes collectively referred to as SLRM). At the anterior-most point in which the DG clearly fills the centre of the lateral portion of the hippocampus, the VHS surrounds the DG (Figure 6(b) and (e)). It extends from the lateral extent of the uncul sulcus (see ‘^’ in Figure 6(b)), underneath the DG and curves in a dorsal direction to extend along the entire lateral edge of the DG (note we have elected to use the spelling ‘uncul’ rather than ‘uncal’ in this protocol). It then curves in a medial direction, extending over the top of the DG. In essence, the anterior portion of the DG is encompassed by the VHS which forms an inverted ‘C’ shape enclosing the ventral, lateral and dorsal extents of the DG (see the black line in Figure 6(e)). Importantly, the VHS forms this characteristic inverted ‘C’ shape at a point where the DG is present within the space it encompasses (Figures 4(b)–6(b) sequentially). We, therefore, use this as an indirect marker of the presence of the DG (note that in left hemisphere, the ‘C’ shape will not be inverted).

Locating the VHS on T2-weighted images is more difficult than locating it on histologically stained tissue. Therefore, before proceeding to locate the VHS on the MRIs, take some time to become familiar with the VHS on the histologically stained tissue presented in ‘b’ and ‘c’ of Figures 3–15 and observe how it changes along the anterior–posterior axis of the hippocampus (which will be described in the following sections). Once you feel confident that you can identify the VHS on the histological slices presented, proceed to the T2-weighted images.

#### Applicability to T2-weighted images

The fattening of the lateral portion of the hippocampus is helpful in determining the approximate location of the anterior-most slice in which the DG is present and can easily be seen on T2-weighted images (see Figures 3(d)–6(d) sequentially – note the gradual fattening of the lateral external digitation of the hippocampus).

The VHS is a stable landmark along the anterior–posterior axis of the hippocampus and can be useful in determining the location of the DG in its entirety. On T2-weighted images, the VHS appears as a thin band of darker voxels in the centre of the lateral portion of the hippocampus (e.g. see the band of darker voxels forming a ring in the centre of lateral portion of the hippocampus in Figure 7(a)). The VHS has been used to delineate the DG in a number of previous imaging studies (Iglesias et al., 2015; Kulaga-Yoskovitz et al., 2015; Wood et al., 2015).

It is important to create a mental framework for the likely location of the VHS along the longitudinal axis of the hippocampus on T2-weighted images. The histologically stained tissue presented in ‘c’ of Figures 3–15 mirrors the signal intensity seen on the T2-weighted images presented in ‘d’ of Figures 3–15. In both cases, grey matter appears lighter and white matter appears darker. It may be helpful to compare ‘c’ and ‘d’ in Figures 3–15 to develop a feel for how the location of the dark band of the VHS as seen on T2-weighted images relates to the location of the VHS on histologically stained tissue along the axis of the hippocampus.

We acknowledge that the VHS can be difficult to see on T2-weighted images and may not be clearly discernible on every slice. For example, the slice in Figure 6(a) is one where the VHS is difficult to see. The ventral and lateral portions of the band of the VHS can be seen relatively clearly, but the dorsal portion of the band is more ambiguous. The slice in Figure 7(a) is one where the ventral, lateral and dorsal extents of the band can more clearly be seen. Patient diligence and careful scrutiny of the patterns of intensity change are required to locate the VHS in each slice. Once confident in the identification of the VHS on T2-weighted images, proceed to Step 1.

##### **Step 1**: identify the anterior-most slice containing the DG

The first step in creating the DG/CA4 mask is to identify the anterior-most slice in which the DG is likely to fill the centre of the lateral portion of the hippocampus. To do this, start at the anterior-most slice of the hippocampus and slowly scroll through images in a posterior direction. As described above, the lateral portion of the hippocampus begins to fatten when moving posteriorly. Prior to this fattening, the VHS can be seen as a relatively straight band of darker voxels in the centre of the hippocampus (Figures 3(d) and 4(d)). Soon after this fattening begins, the lateral end of the VHS can be seen to curve and extend in a dorsal direction (see ‘>’ in Figure 5(d)). Importantly, on histologically stained tissue, at the point that the VHS begins to extend dorsally, the DG is not yet clearly visible (Figure 5(b) and (e)). Moving a few slices posterior to this, the dorsal-most portion of the VHS continues to extend medially creating the inverted ‘C’ shape described above (‘>’ in Figure 6(d)). On histologically stained tissue, it is from this approximate point that the DG can clearly be seen occupying the space within the inverted ‘C’ (Figure 6(b) and (e)). We suggest, therefore, that the anterior-most slice in which the VHS can be seen to form this inverted ‘C’ on T2-weighted images is the slice in which to begin delineating the DG.

Of note, at the resolution of these T2-weighted images, no clear, reliable anatomical markers are available to discern the true anterior-most point of the DG. It is unavoidable that portions of the DG will be present in sections immediately anterior to the point where the inverted ‘C’ is present (e.g. see the portion of DG present in Figure 5(b)). However, these slices are likely to contain a mix of subregions which cannot be differentiated at this resolution (see the unmasked portion in the centre of the lateral hippocampus in Figure 5(e)). The goal of this initial step is to use a reliable neuroanatomical landmark to capture the first slice in which the DG/CA4 is likely to be the dominant structure in the centre of the lateral portion of the hippocampus. We suggest that the anterior-most slice in which the VHS can be seen to form an inverted ‘C’ serves this purpose well and can be seen consistently across participants.

##### **Step 2**: trace the first slice of the DG/CA4 mask

Tracing the DG/CA4 involves two important stages: tracing the VHS, which results in a partial encircling of the DG, and then picking a landmark with which to close the partial circle.

A useful starting point for tracing the VHS is the lateral extent of the uncul sulcus (see and compare the position of ‘^’ in Figure 6(b)–(d)). This may be readily apparent on T2-weighted images as a small collection of brighter voxels which represent cerebrospinal fluid (CSF) (indicated in Figure 6(d)). Place the pointer on the lateral extent of the uncul sulcus (‘^’ in Figure 6(d)) and trace along the dark line of the VHS in a lateral direction. Continue tracing as the line turns in a dorsal direction and then again when it turns in a medial direction. The demarcation for the medial extent of this border may be unclear. On this first slice, we recommend continuing the border until directly above the point at which you started (see position of ‘>’ in Figure 6(d)). When reaching this point, the newly created border should clearly resemble an inverted ‘C’ shape (see the black line in Figure 6(e)–(g)). This inverted ‘C’ serves as the ventral, lateral and dorsal borders of the DG/CA4 mask. Note that before tracing the inverted ‘C’ component of this mask, you must be mindful to leave room for CA1 laterally and CA3/2 dorsally (see Figure 6(g)). To complete the border, place the pointer at the end of the top ‘blade’ of the inverted ‘C’ (‘>’ in Figure 6(d)) and draw a line in a ventral direction until you meet the end of the bottom blade of the inverted ‘C’ (‘^’ in Figure 6(d)). This line serves as the medial border of the DG/CA4 mask. After completing this line, a roughly circular boundary encompasses the centre of the lateral portion of the hippocampus (see the red mask in Figure 6(g)). It is within this space that the DG resides. To complete this first slice of the DG/CA4 mask, fill in the space encircled by the boundary.

### From the first slice of the DG/CA4 mask to the final slice of the uncus

The next step is to repeat the process described in Step 2 above for each subsequent slice in a posterior direction. However, as we move posteriorly, anatomical changes occur along the anterior–posterior axis of the hippocampus which require some adjustments in the method of tracing the DG/CA4 mask.

#### Histology

The first change in anatomy relates to the VHS. At the level of the first slice of the DG/CA4 mask described above, the VHS forms an inverted ‘C’ shape encompassing the DG. However, when moving in a posterior direction, the portion of the VHS overlying the DG gradually recedes in a lateral direction (Figures 6(b)–8(b) sequentially – note the lateral movement of the ‘>’ mark indicating the medial extent of the dorsal portion of the VHS). By the final slice of the uncus, the VHS extends only a short way over the top of the DG (Figure 11(b)).

The second change as we continue posteriorly relates to the lateral extent of the uncul sulcus. At the level of the first slice of the DG/CA4 (Figure 6), the lateral extent of the uncul sulcus sits in a narrow fissure which terminates at the ventromedial base of the lateral portion of the hippocampus (‘^’ in Figure 6(b)). When moving posteriorly, the uncus gradually splits from the lateral hippocampus and as it does, the lateral extent of the uncul sulcus progressively expands and ascends in a dorsal direction (see the ascension of ‘#’ in Figures 8(b)–10(b) sequentially). At the point where the uncus completely separates from the body of the hippocampus, the fimbria gains its characteristic bulbous shape when viewed on coronal sections (see ‘%’ in Figure 11(b) and (c)).

Therefore, between the first slice of the DG/CA4 mask and the final slice of the uncus, the lateral movement of the dorsal extent of the VHS and the expansion of the lateral extent of the uncul sulcus must be taken into account when tracing the DG/CA4 mask.

#### Applicability to T2-weighted images

The lateral movement of the dorsal extent of the VHS can be seen on T2-weighted images, marked with ‘>’ in Figures 6(d)–8(d). As alluded to earlier, a definitive cut-off point for the dorsal-most end of the VHS can be difficult to see. It can, however, generally be estimated with careful scrutiny of the patterns of intensity change.

The expansion of the lateral extent of the uncul sulcus can be seen as a collection of lighter voxels (CSF) on T2-weighted images. When moving in a posterior direction, these lighter voxels progressively appear at the lateral extent of the uncul sulcus (Figure 5(d)) and expand in a dorsal direction as the uncus gradually splits from the lateral hippocampus (note the expansion and dorsal progression of bright voxels in the lateral extent of the uncul sulcus in Figures 7(d)–9(d) sequentially). The bright voxels which indicate its ascension may not be clear on all slices, and in some subjects they may be sparse. In addition, this space can, conversely, appear darker than the surrounding tissue due to the presence of blood vessels which appear as black voxels (see Figure 10(d) for an example). However, it can generally be made out with careful scrutiny of the patterns of intensity change and with a thorough knowledge of and reference to the underlying neuroanatomy.

The expansion of the uncul sulcus continues until the uncus splits from the hippocampus. It is at this point that the fimbria emerges. On T2-weighted images, the fimbria can be seen as a collection of dark voxels sitting dorsomedial to the hippocampal body (see ‘%’ in Figure 11(d) and (g)). Of note, as the uncul sulcus expands, the dorsal-most point of the lateral uncul sulcus naturally becomes the base of the fimbria (compare the position of ‘#’ in Figures 8(d)–11(d) sequentially).

##### **Step 3**: trace from the first slice of the DG/CA4 mask to the final slice of the uncus

The anatomical changes mentioned above should be incorporated into the method of tracing the DG/CA4 between the first slice of the DG/CA4 mask (created in Step 2) and the final slice of the uncus as follows.

For each slice immediately posterior to the first slice of the DG/CA4 mask, the method described in Step 2 is maintained. First, create a border by tracing the inverted ‘C’ of the VHS (see the black line in Figure 7(e)–(g)). Then close this boundary by drawing a line from the end of the top blade of the ‘C’ to the end of the bottom blade (see the red line in Figure 7(g)). Importantly, when moving posteriorly, pay careful attention to the lateral movement of the dorsal portion of the VHS described above. As the top blade of the inverted ‘C’ recedes in a lateral direction, the line closing the boundary between the top and bottom blades of the ‘C’ gradually becomes more oblique (see the gradual lateral rotation of the red line in Figures 6(g)–9(g) sequentially).

In parallel, moving posteriorly, the lateral extent of the uncul sulcus expands in a dorsal direction. From the point that this begins to occur (described above), we recommend a small change in the method of tracing the DG/CA4 boundary. For each slice, start at the ventrolateral extent of the uncul sulcus where the VHS begins (‘^’ in Figures 8(d)–10(d) sequentially) and trace along the VHS until reaching its dorsal-most extent (‘>’ in Figures 8(d)–10(d) sequentially) creating the inverted ‘C’-shaped boundary. Next, from the end of the top blade of the ‘C’ draw a line to the dorsolateral extent of the uncul sulcus (‘#’ in Figures 8(d)–10(d)). Finally, close the boundary by tracing down the medial wall of the hippocampus until reaching the start point at the end of the bottom blade of the ‘C’ (the orange line in ‘e’, ‘f’ and ‘g’ in Figures 8–10). This final line will be very short in more anterior slices (see the orange line in Figure 8(g)) and lengthen in more posterior slices as more of the medial wall of the hippocampus is exposed (see progressive lengthening of the orange line in Figures 8(g)–10(g) sequentially).

By the point that the fimbria begins to emerge, the dorsolateral extent of the uncul sulcus meets the fimbria (see and compare location of ‘#’ in Figures 10(d) and 11(d)). Accordingly, from the point that the fimbria begins to emerge, first trace the VHS and then draw a line from the top blade of the inverted ‘C’ to the ventromedial base of the fimbria (labelled ‘#’ in Figure 11(d)). Then trace down the medial wall of the hippocampus until reaching the start point at the end of the bottom blade of the inverted ‘C’ (the orange line in Figure 11(g)). Continue using this method to trace the DG/CA4 mask up to and including the final slice of the uncus. The final slice of the uncus can be seen as a small collection of grey voxels at the posterior-most portion of the uncus, medial to the lateral portion of the hippocampus (see the purple region in Figure 11(g)). This is described in more detail in Part 6: the uncus.

### From the final slice of the uncus to the tail of the hippocampus

We next explain important changes in anatomy which occur between the final slice of the uncus and the tail of the hippocampus and then describe the resulting changes in the method of tracing the DG/CA4 mask between these points.

#### Histology

Between the final slice of the uncus and the tail of the hippocampus, the body of the hippocampus is quite consistent in its anatomy. However, the hippocampus (and so the dorsal extent of the VHS) continues to rotate in a lateral direction (see Figures 11(b)–13(b) sequentially – note the lateral movement of ‘>’ indicating the dorsal extent of the VHS). This rotation must continue to be incorporated into the DG/CA4 mask and is the only significant change that occurs until reaching the tail of the hippocampus.

When moving into the tail, the fimbria begins to elongate (‘%’ in Figure 13(b)) and gradually loses the characteristic bulbous shape seen in more anterior slices. It is from approximately this point that it is referred to as the fornix. In parallel, the lateral portion of the hippocampus containing the DG also begins to elongate in a dorsal direction (Figure 13(b)). This elongation of the lateral portion of the hippocampus and DG must be incorporated into the DG/CA4 mask.

When moving more posteriorly, the fimbria disappears, leaving the elongated lateral portion of the hippocampus (Figure 14(b)). At this level, the VHS can be seen to close and form an undulating circle (Figure 14(b) and (c)). This circle encompasses the posterior-most portion of the DG/CA4 (see the location of DG in Figure 14(b)).

#### Applicability to T2-weighted images

Each of the markers described in the previous section can be seen on T2-weighted images. For instance, note the ventrolateral movement of the dorsal extent of the VHS, marked with a ‘>’ in Figures 11(d)–13(d). The elongation of the fimbria is also clearly visible (‘%’ in Figure 13(d)), as is the elongation of the lateral portion of the hippocampus (see the elongation between Figures 12(d) and 13(d)). More posteriorly, the undulating circle of the VHS is also apparent (see the dark ring of the VHS in Figure 14(d)). This characteristic undulating circle is important for locating the final slice in which the DG is likely to be present on T2-weighted images.

##### **Step 4**: trace from the final slice of the uncus to the tail of the hippocampus

The method for tracing the DG/CA4 mask described in Step 3 is maintained from the final slice of the uncus until the point that the crus of the fornix appears in the tail of the hippocampus. From the final slice of the uncus, the VHS continues to rotate in a lateral direction. It is important to remain faithful to this rotation when tracing the VHS.

For each slice until the point that the crus of the fornix appears, first, trace the VHS and then draw a line from the top blade of the inverted ‘C’ to the ventromedial base of the fimbria (see the red line in Figure 12(g)). This also pertains to more posterior slices in which the fimbria is elongated. Then trace down the medial wall of the hippocampus until reaching the start point at the end of the bottom blade of the inverted ‘C’ (the orange line in Figures 11(g)–13(g)). Note that from the point that the fimbria elongates to become the fornix, the body of the hippocampus also elongates. The entire length of the body of the hippocampus must be incorporated in the DG/CA4 mask.

Moving posteriorly, as the fornix gradually disappears and becomes the dorsolateral wall of the hippocampus, the VHS can be seen to ‘close’, creating the characteristic undulating circle described in the previous section. This ring encompasses the posterior-most portion of the DG. We recommend using the final slice in which this ring of dark voxels encompasses lighter voxels (indicating grey matter) as the final slice of the DG/CA4 mask (Figure 14(g)). Simply trace around the dark ring of the VHS being careful to leave a space for the CA3/2 dorsolaterally and the CA1 and subiculum ventrolaterally (Figure 14(g)).

It is important to reiterate that when moving from anterior to posterior, the hippocampus gradually rotates in a lateral direction. This, in turn, results in a gradual lateral rotation of the VHS and DG along the longitudinal axis of the hippocampus. In more anterior slices, tracing the VHS results in an inverted ‘C’. By the tail of the hippocampus, the ‘C’ has rotated in a lateral direction to such a degree that it resembles more of a ‘U’ (see the black line in Figures 6(e)–13(e) sequentially – note the inverted ‘C’ in Figure 6(e) rotates to become a more akin to a ‘U’ by Figure 13(e)). The rotation of the hippocampus along its longitudinal axis must be accommodated when creating the DG/CA4 mask, as the masks for other CA regions will be created in relation to the orientation and shape of the DG/CA4 mask. When the method described above is reproduced on coronal sections of histologically stained tissue, the entire DG is encompassed within the resulting mask which also incorporates the gradual rotation of the DG along the longitudinal axis (Figures 6(e)–14(e)).

## Part 2: the CA3/2 mask

CA3 and CA2 are located dorsal to the DG. While some authors have utilised geometric rules to create separate CA2 and CA3 masks at 4T (Mueller et al., 2007), the boundary between CA3 and CA2 cannot be reliably identified on MRI scans at this resolution, in our opinion. These regions are therefore grouped together (Figure 1(d)).

### First slice of the CA3/2 mask

There are no reliable markers with which to definitively identify the anterior-most slice containing the CA3/2 on T2-weighted images. We must, therefore, rely on knowledge of the underlying neuroanatomy for clues with which to identify a likely starting point for this mask.

#### Histology

Ding and Van Hoesen (2015) recently conducted a thorough immunohistochemical investigation of the head of the human hippocampus. In serial sections of histologically stained hippocampal tissue, they reveal that the anterior-most point of CA2 gradually emerges from CA1. Moving further posteriorly, the CA3 emerges adjacent to the CA2 and, as it does, the CA2 recedes in a lateral direction.

CA1 predominates in the lateral portion of the anterior hippocampus prior to the emergence of the DG (Figures 3(e)–5(e)). CA1 surrounds the dorsal, lateral and ventral portions of the DG at the point that it first begins to emerge (Figure 5(e)). As observed by Ding and Van Hoesen (2015), moving posteriorly, CA2 begins to blend with the CA1 until finally CA2 predominates and overlies the DG (Figure 6(e)). Importantly, the gradual transition from CA1 to CA2 occurs in parallel with the emergence of the DG to fill the space within the inverted ‘C’ of the VHS. Therefore, in slices immediately anterior to the emergence of the inverted ‘C’, the cortical strip above the dorsal extent of the VHS is likely to contain a mix of CA1 and CA2 or more anteriorly only CA1. It is after the dorsal extent of the VHS clearly extends medially and the DG fills the space enclosed by the inverted ‘C’ that CA2 predominantly overlies the DG (Figure 6(e), and see Ding and Van Hoesen, 2015).

In brief, the anterior-most slice in which the VHS can be seen to form an inverted ‘C’ is an important anatomical marker with which to locate the anterior-most slice in which the CA3/2 is likely to be present without being overly contaminated by the presence of other subregions. Therefore, we recommend creating the anterior-most slice of the CA3/2 mask at this point. Of note, the first slice of the DG/CA4 mask is also created on this slice.

In relation to useful landmarks with which to create the borders of the CA3/2 mask in this first slice, the ventral border of CA2 runs along the dorsal portion of the VHS (Figure 6(e)). The dorsal border is the superior hippocampal wall. The medial border lies approximately at the medial extent of the lateral-most external digitation of the hippocampus (Ding and Van Hoesen, 2015) (see ‘!’ in Figure 6(b)). CA2 curves in a ventromedial direction at this point and transitions into the CA1 region of the uncus (see Ding and Van Hoesen, 2015). The lateral extent of CA2 can be observed on histologically stained tissue at the point where the thin layer of neurons comprising CA2 fans out to become the slightly thicker layer of neurons comprising CA1 (see Figure 6(b)). Importantly, this transition occurs at a position dorsolateral to the DG at the point where the dorsolateral wall of the hippocampus turns ventrally. This transition consistently occurs in this position along the entire longitudinal axis of the hippocampus. The first slice of the CA3/2 mask, therefore, fills the entire dorsal portion of the lateral hippocampus (see the green region in Figure 6(e)).

Before moving to the T2-weighted images, it is good practice to develop a clear understanding of where the CA3/2 lies on coronal sections of histologically stained tissue, especially in relation to its lateral border. Importantly, the lateral border of CA2 (denoting the transition between CA2 and CA1) cannot be seen on T2-weighted images. We recommend careful study of the location of the CA2–CA1 transition on histologically stained tissue along the longitudinal axis of the hippocampus before attempting to delineate this region on MRIs.

#### Applicability to T2-weighted images

Useful landmarks to delineate the ventral, dorsal and medial borders of CA2 in this first slice are the dorsal portion of the VHS (see ‘>’ in Figure 6(d)), the superior hippocampal wall and the medial extent of the lateral-most external digitation of the hippocampus, respectively (see ‘!’ in Figure 6(d)). Each of these can be seen on T2-weighted images. As mentioned in the previous section, the transition between CA2 and CA1 is not apparent on MRI. There are no consistent intensity changes between these regions. Therefore, knowledge of the underlying neuroanatomy must be used to decide on the likely location of the lateral border of the CA3/2 mask. As noted above, the transition between CA2 and CA1 consistently occurs at a position dorsolateral to the DG at the point where the dorsolateral wall of the hippocampus turns ventrally. Based on this consistency as observed on histologically stained tissue, we can infer the likely location that this transition is expected to occur on MRI. A method for doing this is described in Step 5.

##### **Step 5**: create the first slice of the CA3/2 mask

This step should be done after creating the DG/CA4 mask. To create the first slice of the CA3/2 mask, first, scroll to the slice containing the anterior-most slice of the DG/CA4 mask. The CA3/2 mask will be created in the strip of cortical tissue overlying the DG/CA4 mask (see the green region in Figure 6(g)).

Begin by tracing the lateral border of the CA3/2 mask. To do this, place the pointer on a position at the dorsolateral ‘corner’ of the DG/CA4 mask (see ‘1’ in Figure 6(g)) and then draw a straight diagonal line to the dorsolateral ‘corner’ of the superior wall of the hippocampus (see the green line in Figure 6(g)). Then trace along the superior wall of the hippocampus in a medial direction until reaching the medial extent of the lateral-most external digitation of the hippocampus (see ‘!’ in Figure 6(d)). From this point, draw a straight line in a ventrolateral direction until reaching the dorsomedial extent of the VHS (i.e. the end of the top blade of the inverted ‘C’; see ‘>’ in Figure 6(d)). Then to close the mask trace along the VHS following the dorsal border of the DG/CA4 mask in a lateral direction until reaching the starting point at the dorsolateral ‘corner’ of the DG/CA4 mask. Finally, fill in the space enclosed by the newly created boundary.

### From the first slice of the CA3/2 mask to the final slice of the uncus

The next step is to repeat the process described in Step 5 for each subsequent slice in a posterior direction. However, as we move posteriorly, anatomical changes occur along the anterior–posterior axis of the hippocampus which require an adjustment to the method of tracing the CA3/2 mask.

#### Histology

Between the first slice of the CA3/2 mask and the final slice of the uncus, the only change in anatomy that impacts the tracing method relates to the medial extent of the lateral-most external digitation of the hippocampus. At the level of the first slice of the CA3/2 mask, the medial extent of the lateral-most digitation lies in a depression (see ‘!’ in Figure 6(b)). Moving posteriorly, the medial/uncul portion of the hippocampus fattens and, as it does, this depression begins to fill and push dorsally (see location of ‘!’ in Figures 6(b)–10(b) sequentially), with this depression becoming the dorsolateral-most point of the fimbria (see ‘!’ in Figure 11(b)). Between the first slice of the CA3/2 mask and the final slice of the uncus, the changing nature of the depression which indicates the medial extent of the lateral-most external digitation of the hippocampus and the emergence of the fimbria must be taken into account.

#### Applicability to T2-weighted images

The ascension of the medial extent of the lateral external digitation and the emergence of the fimbria can both be seen on T2-weighted images. The depression of the medial extent of the lateral external digitation is usually clear in the majority of participants (note the location of ‘!’ in Figures 6(d)–11(d) sequentially). Locating the fimbria on T2-weighted images was discussed in Part 1: the DG/CA4 mask.

##### **Step 6**: trace from the first slice of the CA3/2 mask to the final slice of the uncus

The method for tracing the CA3/2 mask described in Step 5 is maintained from the first slice of the mask until the point at which the uncus begins to split away from the lateral portion of the hippocampus. For each slice until this point, first, trace the lateral border of the CA3/2 mask by drawing a diagonal line from the dorsolateral ‘corner’ of the DG/CA4 mask to the dorsolateral ‘corner’ of the superior wall of the hippocampus (the green line in Figures 6(g)–11(g)). Then trace along the superior wall of the hippocampus in a medial direction until reaching the medial extent of the lateral external digitation (see ‘!’ in Figure 6(d)-10(d)). Note that when moving posteriorly, this point will gradually move in a dorsal direction. From here, draw a line to the dorsomedial extent of the VHS. Then trace along the dorsal border of the DG/CA4 mask until reaching the starting point. Finally, fill in the space enclosed by the newly created boundary.

Moving posteriorly, the uncus splits from the hippocampus and the fimbria emerges. From this point, the method of tracing the dorsal and medial borders of the CA3/2 mask undergoes a slight change. From where the fimbria begins to appear, first trace the lateral border of the CA3/2 mask as described above. Then trace the dorsal border along the superior hippocampal wall until reaching the medial extent of the lateral external digitation (see ‘!’ in Figure 11(d)). Note that this now sits at the dorsolateral base of the fimbria. From here, draw a line in a ventromedial direction, cutting through the base of the fimbria until reaching the dorsomedial ‘corner’ of the DG/CA4 mask (see Figures 10(g) and 11(g)). Then trace along the dorsal border of the DG/CA4 mask until reaching the starting point and fill in the space enclosed by the newly created boundary. Therefore, from the point that the fimbria begins to emerge, the medial wall of the CA3/2 mask is the base of the fimbria. Continue with this method up to and including the final slice of the uncus. The final slice of the uncus can be seen as a small collection of grey voxels at the posterior-most portion of the uncus, medial to the lateral portion of the hippocampus (see the purple region in Figure 11(g)). This is described in more detail in Part 6: the uncus.

### From the final slice of the uncus to the tail of the hippocampus

The method described in Step 6 should be applied along the body of the hippocampus. However, careful attention must be paid to the lateral rotation of the hippocampus, which should be taken into account when determining the location of the lateral border of the CA3/2 mask. This border must be maintained at the dorsolateral ‘corner’ of the DG/CA4 mask along the longitudinal axis of the hippocampus. Therefore, moving posteriorly, as the DG/CA4 mask gradually rotates in a lateral direction, the lateral border of the CA3/2 mask will also gradually move in a ventrolateral direction keeping in alignment with the rotation of the DG/CA4 mask (compare the lateral border of the CA3/2 mask in Figures 6(g)–13(g)). Note that a number of important anatomical changes occur from the point that the crus of the fornix appears. These are described in the following sections.

#### Histology

Shortly after the crus of the fornix appears (see ‘%’ in Figure 13(b)), two separate portions of the hippocampus are visible. The ‘typical’ ventral portion can be seen at the ventral extent of the fornix and a dorsal portion appears at the dorsal end of the fornix (see Figure 13(b); a portion of the DG and what is likely to be CA3/2 can be seen at the dorsal end of the fornix). Moving further posteriorly, the ventral portion of the hippocampus becomes elongated, its dorsal end extending along the fornix until it joins with the dorsal portion. As this occurs, the fornix disappears, becoming the dorsolateral wall of the hippocampus (Figure 14(b)). From this point, the posterior-most portion of the hippocampus can be seen as an ovoid shape (Figure 14(b)). Final remnants of the DG can be seen in the centre of the ovoid hippocampus and a thin layer of cells can be seen running along the dorsolateral wall of the hippocampus (Figure 14(b)). This thin layer of cells is architecturally similar to the thin layer of CA3/2 cells in more anterior slices (see this in Figures 6(b)–13(b)). This thin layer of cells ‘fans out’ at each end of the ovoid hippocampus (indicated by the green lines in Figure 14(e)) in a similar manner to the fanning out that occurs at the CA2–CA1 border in more anterior sections (compare with fanning out of the cell layer in Figures 6(b)–13(b)). Our interpretation of this is that the dorsolateral portion of the ovoid hippocampus contains a thin layer of CA3/2 cells which fan out at both the dorsomedial and ventrolateral ends of the hippocampus to become dorsal and ventral portions of the CA1, respectively (see the position of the green lines in Figure 14(e) and then see the fanning out of the cell layer at the equivalent points in Figure 14(b)).

The thin layer of cells sitting along the dorsolateral wall of the hippocampus, which we interpret as being CA3/2, becomes thicker when moving in a posterior direction (compare cell layer thickness in Figures 14(b) and 15(b)). We argue that the well-characterised fanning out that occurs between the CA2 and CA1 in a medial–lateral direction as seen on coronal sections also occurs in an anterior–posterior direction in this posterior portion of the hippocampus. In short, the thin layer of CA3/2 cells seen in the dorsolateral portion of the hippocampus (Figure 14(b)) fans out in a posterior direction to become a layer of CA1 cells (Figure 15(b)). This transition occurs from the approximate point that the posterior-most portion of the DG can be seen (Figure 14(b)). We, therefore, suggest that the posterior-most portion of the DG may be a useful marker for the final slice of the CA3/2 mask.

#### Applicability to T2-weighted images

The crus of the fornix can be seen on T2-weighted images as a dark band of tissue extending from the dorsal wall of the hippocampus in a dorsomedial direction (see ‘%’ in Figure 13(d)). The emergence of the dorsal portion of the hippocampus at the dorsal end of the fimbria can be seen as an expanding collection of lighter voxels (Figure 13(d)). The elongation of the ventral portion of the hippocampus can also be clearly seen (compare the ‘typical’ ventral portion of hippocampus in Figures 12(d) and 13(d)). Moving further posteriorly, the posterior-most portion of the hippocampus which, as mentioned earlier in this Part, has an ovoid shape is visible (Figures 14(d) and 15(d)).

##### **Step 7**: trace from the final slice of the uncus to the tail of the hippocampus

The method for tracing the CA3/2 mask described in Step 6 is maintained from the final slice of the uncus until reaching the tail of the hippocampus. Moving posteriorly, it is important to keep the lateral border of the CA3/2 mask in alignment with the dorsolateral ‘corner’ of the DG/CA4 mask as it rotates in a lateral direction. This results in the lateral border of the CA3/2 mask progressively moving in a ventrolateral direction (note ventrolateral movement of the lateral border of the CA3/2 mask with rotation of the DG/CA4 mask in Figures 11(g)–13(g) sequentially).

From the point that the crus of the fornix appears, an alteration in the tracing method is required. First, trace the lateral border of the CA3/2 mask as described in Step 6. Then trace the dorsal border along the superior hippocampal wall until reaching the fornix. Continue tracing along the ventromedial wall of the fornix until reaching the DG/CA4 mask (Figure 13(g)). From here, trace along the dorsal border of the DG/CA4 mask in a ventrolateral direction until reaching the starting point and then fill in the space enclosed by the newly created boundary. Continue in this way as the ventral hippocampus expands in a dorsal direction until the point at which the fornix becomes the dorsolateral wall of the hippocampus. Note that the CA3/2 mask becomes elongated with the elongation of the ventral hippocampus (see Figures 12(g)–14(g)).

From the point at which the fornix becomes the dorsolateral wall of the hippocampus, the VHS begins to start forming an undulating circle which encompasses the DG. This is described in Part 1: the DG/CA4 mask. As this occurs, a thin band of grey matter can be seen between the dorsolateral wall of the VHS and the dorsolateral wall of the hippocampus (see Figure 14(d)). This corresponds to the band of thin cells described earlier in this Part which we speculate contains CA3/2. To accommodate this, first trace the lateral border of the CA3/2 mask as described in Step 6. Then trace along the dorsolateral wall of the hippocampus in a dorsomedial direction and continue tracing until reaching a point at the dorsomedial end of the ovoid hippocampus (see ‘9’ in Figure 14(g)). From here, draw a line in a ventrolateral direction until reaching the DG/CA4 mask. Then trace along the dorsal border of the DG/CA4 mask in a ventrolateral direction until reaching the starting point and then fill in the space enclosed by the newly created boundary. Continue with this method until the slice which contains the final slice of the DG/CA4 mask. However, moving posteriorly, the length of the CA3/2 mask will become smaller. As a general rule, the length of the CA3/2 mask in these most posterior slices should approximately mirror the length of the DG/CA4 mask. It is from around the point that the DG ends that the CA3/2 transitions to CA1 in a posterior direction. Therefore, the slice which contains the final slice of the DG/CA4 mask will also contain the final slice of the CA3/2 mask.

## Part 3: the CA1 mask

CA1 is located lateral to CA2/3 and the DG.

### First slice of the CA1 mask

#### Histology

As with the CA3/2 mask, locating the anterior-most slice in which the CA1 is definitively present is difficult. As noted by Ding and Van Hoesen (2015), when viewed in serial sections of histologically stained hippocampal tissue from anterior to posterior, the anterior-most point of the CA1 gradually emerges from the prosubiculum and then expands to fill the lateral portion of the anterior hippocampus (Figures 4(e) and 5(e)). CA1 is first seen at the anterior-most point that the lateral external digitation of the hippocampus begins to bend in a dorsal direction (Ding and Van Hoesen, 2015) (see ‘!’ in Figure 3(b)). Therefore, the slice where this is first observed delineates the anterior-most slice of the CA1 mask.

#### Applicability to T2-weighted images

The point at which the lateral external digitation of the hippocampus first begins to bend in a dorsal direction can be seen on T2-weighted images (see ‘!’ in Figure 3(d)) and can be used as a marker for the anterior-most slice of the CA1. Another important landmark is the VHS. At this level, the VHS can be seen as a darker band of voxels running along the centre of the thin ribbon of the hippocampus (Figures 3(d) and (g)). This may be difficult to see in some participants.

##### **Step 8**: create the first slice of the CA1 mask

First, identify the anterior-most slice in which the lateral external digitation of the hippocampus begins to bend in a dorsal direction. To do this, start at the anterior-most slice of the hippocampus and scroll through images in a posterior direction.

Begin by creating the dorsomedial border of the CA1 mask. To do this, place the pointer on the dorsal wall of the hippocampus in the centre of the shallow depression created by the bend (see ‘!’ in Figure 3(d)). From here, draw a straight line in a ventral direction until reaching the VHS (see the purple line in Figure 3(g)). Then draw a diagonal line in a ventrolateral direction until reaching the darker voxels which indicate white matter (see the blue line in Figure 3(g)). From this point, trace along the grey–white matter junction in a lateral direction and along the ventral wall of the hippocampus. Continue tracing around the curve of the lateral extent of the hippocampus and along the dorsal wall of the hippocampus until reaching the starting point in the centre of the shallow depression created by the bend on the dorsal wall of the hippocampus. Finally, fill in the space enclosed by the newly created boundary.

### From the first slice of the CA1 mask to the final slice of the uncus

The next step is to repeat the process described in Step 8 for each subsequent slice in a posterior direction. However, as we move posteriorly, anatomical changes occur along the anterior–posterior axis of the hippocampus which require an adjustment to the tracing method.

#### Histology

Moving posteriorly from the first slice of the CA1 mask, the lateral portion of the hippocampus begins to fatten resulting in an expansion of the lateral external digitation of the hippocampus (Figures 3(e)–6(e)). As this occurs, the shallow depression created by the slight bend in the dorsal wall of the hippocampus in the first slice progressively becomes a deeper depression (see ‘!’ in Figures 3(b)–6(b)). In parallel, the VHS progressively extends in a dorsal direction (compare the location of ‘>’ in Figures 4(b) and 5(b)). As the lateral portion of the hippocampus fattens and the VHS extends dorsally, CA1 occupies the lateral and dorsal portions of the lateral hippocampus (Figure 3(e)–5(e)).

Moving posteriorly, however, the portion of the CA1 residing in the dorsal part of the lateral hippocampus gradually transitions to become the CA2/3. As mentioned previously, this transition occurs at the approximate point where the DG begins to emerge (Ding and Van Hoesen, 2015). By the point that the DG has fully emerged to occupy the space enclosed by the inverted ‘C’ of the VHS, CA1 has receded to occupy only the lateral wall of the lateral hippocampus (Figure 6(e); see also Ding and Van Hoesen, 2015). It is important to note that moving posteriorly, the transition between CA1 and CA2/3 is gradual. As a result, the exact transition point cannot be established on MRI at this resolution. Moving further posteriorly, CA1 occupies the cortical ribbon adjacent to the lateral wall of the lateral hippocampus along its longitudinal axis until reaching the tail of the hippocampus (Figures 6(e)–13(e)).

In relation to the borders of CA1 on the coronal plane, in slices anterior to the first slice of the CA3/2 mask, the dorsomedial border is the medial extent of the lateral external digitation (see the purple line in Figures 3(e)–4(e) and the dorsal blue line in Figure 5(e)). After the emergence of the CA3/2 mask, the dorsomedial border of the CA1 mask is the lateral border of the CA3/2 mask (see the green line in Figures 6(e)–14(e)). The ventral border of CA1 occurs at the transition between CA1 and the prosubiculum. On histology, this is apparent at the point where the layer of CA1 neurons fans out to become the thicker band of neurons of the prosubiculum and subiculum (see ‘2’ in Figure 8(b) for example). While this can be difficult to see on some slices, Ding and Van Hoesen (2015) recently provided cyto- and chemo-architectural evidence for the location of the CA1–subiculum border. They found that this border consistently occurs at a point where the lateral wall of the hippocampus bends in a ventromedial direction (see ‘2’ in Figures 3(b)–11(b)). Therefore, this ventrolateral ‘corner’ of the lateral hippocampus is a reliable marker for the approximate location of the transition between CA1 and the subiculum. This location has also been adopted in previous neuroimaging studies (e.g. Iglesias et al., 2015).

#### Applicability to T2-weighted images

Each of the landmarks described above, including the fattening lateral portion of the hippocampus, the medial extent of the lateral external digitation of the hippocampus and the VHS, can be seen on T2-weighted images and have been described in previous steps. The bend at the ventrolateral portion of the lateral hippocampus can also be seen on T2-weighted images (see ‘2’ in Figures 3(d)–11(d)).

##### **Step 9**: trace from the first slice of the CA1 mask to the final slice of the uncus

The anatomical changes mentioned in the previous section should be incorporated into the method of tracing the CA1 between the first slice of the CA1 mask (created in Step 8) and the final slice of the uncus, as follows.

For each slice immediately posterior to the first slice of the CA1 mask, the method described in Step 8 is maintained. First, place the pointer on the dorsal wall of the hippocampus in the centre of the shallow depression created by the bend (see ‘!’ in Figure 4(d)) and draw a straight line in a ventral direction until reaching the VHS (see the purple line in Figure 4(g)). From here, draw a diagonal line in a ventrolateral direction until reaching the ventral wall of the hippocampus (see the blue line in Figure 4(g)). Then trace along the ventral wall of the hippocampus in a lateral direction and continue tracing around the curve of the lateral extent of the hippocampus until reaching the starting point on the dorsal wall of the hippocampus.

As described in Part 1: the DG/CA4 mask, moving posteriorly, the lateral portion of the hippocampus begins to fatten. As this happens, the lateral external digitation of the hippocampus expands resulting in a deepening of the depression at the medial extent of the lateral external digitation (see the depression underlying ‘!’ in Figures 4(d)–6(d) sequentially). As the lateral hippocampus fattens, the VHS extends dorsally into the central space of the lateral hippocampus. The CA1 resides in the cortical ribbon which lies adjacent to the dorsal and lateral walls of the lateral hippocampus (see the blue region in Figure 5(g)). This ribbon can be seen as a band of lighter voxels lying between the dark bands of the VHS and the outer wall of the hippocampus (see Figure 5(a)). This ribbon may be difficult to see on some slices. We recommend the following method to delineate it.

Continue with the method described above until such a point that the VHS begins to expand in a dorsal direction (see Figure 5(d)). From this point, significant changes in the tracing method are necessary. As the VHS expands dorsally, the lateral ribbon of the CA1 can more easily be seen. From this point, for each slice, begin tracing the CA1 mask by placing the pointer on the ventrolateral corner of the lateral hippocampus (see ‘2’ in Figure 5(d)). As described earlier in this Part, this roughly coincides with the transition between the CA1 and subiculum. From here, draw a diagonal line in a dorsomedial direction until reaching the VHS (see the blue line in Figure 5(g)). Then trace along the VHS in a dorsolateral direction until reaching its dorsal-most extent. From this point, draw a straight line to the medial extent of the lateral external digitation (see ‘!’ in Figure 5(d)). Trace around the dorsal wall of the hippocampus in a lateral direction and continue tracing down the lateral wall until reaching the starting point at the ventrolateral corner. Continue this method for each slice in a posterior direction. For each subsequent slice in a posterior direction, take care to note the gradual medial extension of the dorsal portion of the VHS. Carry on with this method until such a point that the inverted ‘C’ of the VHS is created. Here, the tracing method necessarily needs to change one more time.

On the slice in which the inverted ‘C’ of the VHS becomes clear, the DG/CA4 mask and the CA3 mask will already have been created. From this point, the CA1 mask will only occupy the lateral wall of the lateral hippocampus. To trace the CA1 mask on this slice, begin by placing the pointer on the ventrolateral corner of the lateral hippocampus (see ‘2’ in Figure 6(d)). From here, draw a line in a dorsomedial direction until reaching the ventrolateral ‘corner’ of the DG/CA4 mask (see the blue line in Figure 6(g)). Then trace around the DG/CA4 mask border in a dorsolateral direction until reaching the lateral border of the CA3/2 mask. From here, draw a line along the lateral border of the CA3/2 mask until reaching the dorsolateral wall of the hippocampus. Trace around the hippocampal wall in a ventral direction until reaching the starting point of the CA1 mask. This method is continued until the final slice of the uncus. The final slice of the uncus can be seen as a small collection of grey voxels at the posterior-most portion of the uncus, medial to the lateral portion of the hippocampus (see the purple region in Figure 11(g)). This is described in more detail in Part 6.

### From the final slice of the uncus to the tail of the hippocampus

Between the final slice of the uncus and the tail of the hippocampus, no anatomical changes occur which require an alteration in the method described above. Therefore, the method described in Step 9 is applied along the body of the hippocampus. However, careful attention must be paid to the lateral rotation of the hippocampus. This rotation must be taken into account when determining the location of the ventral border of the CA1 mask which undergoes a slight change when moving posteriorly (described in the next section). A number of important anatomical changes occur when entering the tail of the hippocampus and are described in the following section.

#### Histology

Between the final slice of the uncus and the tail of the hippocampus, the CA1 remains in the same location. However, along the body of the hippocampus, the ventromedial extent of the CA1 may extend slightly more medially than it does in more anterior sections. According to Duvernoy et al. (2013), the ventromedial border of the CA1 roughly corresponds to a point which sits half way between the centre and lateral-most extent of the DG. Therefore, the mid-point of the DG may be a useful landmark for delineating the ventromedial extent of the CA1 along the body of the hippocampus.

As described in Part 2: the CA3/2 mask, after the appearance of the crus of the fornix, two separate portions of the hippocampus become visible. The ‘typical’ ventral portion can be seen at the ventral extent of the fornix and a dorsal portion appears at the dorsal end of the fornix (see Figure 13(b)). Moving posteriorly, the ventral portion of the hippocampus expands in a dorsal direction. According to Duvernoy et al. (2013) and Adler et al. (2014), as this expansion occurs, CA1 may also expand in a medial direction and maintain its ventromedial border at roughly the mid-point of the DG (see Figure 13(e)). Therefore, the expansion of the ventral hippocampus is an important marker for modifying the CA1 mask.

As the dorsal and ventral portions of the hippocampus join, the CA1 may continue to expand medially until it dominates the space ventral to the DG (see Figure 14(e)). Moving further posteriorly, CA3/2 that is located dorsolateral to the DG transitions to become CA1 (described in Part 2: the CA3/2 mask) resulting in the posterior-most slices of hippocampus being dominated by CA1 (see Figure 15(e)).

#### Applicability to T2-weighted images

Each of the features which can be used as landmarks described in the previous section can be seen on T2-weighted images. The DG mask has been created in a previous step and can be used to adjust the ventromedial border of the CA1 mask. When moving into the hippocampal tail, as described in the previous section, the expansion of the ventral portion of the hippocampus can be seen (Figure 13(d)) as can the point from which the dorsal and ventral portions are both clear (see Figure 23(g)).

##### **Step 10**: trace from the final slice of the uncus to the tail of the hippocampus

From the final slice of the uncus, the method described in Step 9 is maintained with one alteration. From the final slice of the uncus, create the ventromedial border of the CA1 mask at a point which sits roughly at the centre of the DG/CA4 mask (see the position of the border in Figure 12(g)). Continue this method until reaching the crus of the fornix.

After the appearance of the crus of the fornix, the ventral portion of the hippocampus extends in a dorsal direction (described in Part 2: the CA3/2 mask). From here, continue using the method described in Step 9 to create the ventromedial border in the centre of the DG/CA4 mask. Note that this will result in a gradual elongation of the CA1 mask as the ventral portion of the hippocampus extends dorsally. Continue this method until the fornix becomes the dorsolateral wall of the hippocampus and can no longer be seen.

From the point that the fornix can no longer be seen, the hippocampus begins to take on an ovoid shape and the anatomy becomes much more difficult to see. From this point, designate the cortical ribbon which lies ventromedial to the DG/CA4 mask as CA1. Begin by placing the pointer in line with the medial most point of the DG/CA4 mask (see Figure 14(g) and 23(j)). From here, trace along the ventromedial border of the DG/CA4 mask in a ventrolateral direction until reaching the ventromedial extent of the CA3/2 mask. Trace along the CA3/2 border in a lateral direction until reaching the lateral wall of the hippocampus (see ‘10’ in Figure 14(g)). From here, trace along the wall of the hippocampus in a ventromedial direction and continue tracing along the grey–white matter junction. This may be convoluted and so careful scrutiny is required. Trace along the grey-white matter junction until reaching a point aligned with the starting point. Then, draw a line in a dorsal direction until reaching the starting point (see the blue line in Figure 14(g)). Continue this method until the final slice of the DG/CA4.

Following the final slice of the DG/CA4 mask, we recommend delineating all remaining grey matter in the posterior-most slices of the ovoid hippocampus as CA1. This is in alignment with the delineations of Duvernoy et al. (2013) and Adler et al. (2014). In these final sections of hippocampus, simply trace around the remaining grey matter within the ovoid hippocampus. Remnants of the VHS may still be present here (see Figure 15(d)). If they are, draw around them leaving the space unfilled (see Figure 15(g)). Then fill in the space enclosed within the boundary, being careful not to include the space within the VHS. Continue this method until the final slice in which grey matter can be seen within the shrinking ovoid hippocampus (see progression in Figure 24(b)–(d)).

## Part 4: the subiculum

The subicular cortices occupy the ventral portion of the hippocampus, medial to CA1. They comprise the prosubiculum, subiculum, presubiculum and parasubiculum (see Figure 1(d)). While the border between the subiculum and presubiculum can be inferred by intensity changes on T2-weighted images at this resolution (Ding and Van Hoesen, 2015), the border between the prosubiculum and subiculum cannot be differentiated in these scans. Likewise, the border between the presubiculum and parasubiculum cannot be differentiated. We therefore create two separate subicular masks – a combined prosubiculum/subiculum mask and a combined pre/parasubiculum mask. Here, we first describe a method for creating a mask of the prosubiculum/subiculum region (a method for creating a mask of the pre/parasubiculum is presented later in Part 5). We refer to this collectively as the subiculum mask.

### First slice of the subiculum mask

#### Histology

According to the recent histological examinations of Ding and Van Hoesen (2015), the subiculum is present in the most anterior portions of the hippocampus. Ding and Van Hoesen suggest that within the anterior-most slices, the ventral portion of the hippocampus contains ‘typical’ subiculum (relevant to this step) which, when moving posteriorly, maintains its ventral position along the longitudinal axis of the hippocampus. In contrast, the dorsal portion of the anterior hippocampus contains ‘uncul’ subiculum which, when moving posteriorly, becomes part of the uncus (this mask will be created later in Part 6). Using these observations as a guide, we recommend dividing the anterior-most slices of the hippocampus into ventral (‘typical’ subiculum) and dorsal (‘uncul’ subiculum) portions (see Figure 16(d) and (e)) and creating the first slice of the subiculum mask in the ventral half of the anterior hippocampus.

#### Applicability to T2-weighted images

On T2-weighted images, the anterior-most slice of the hippocampus is small and has an ovoid shape (see Figure 16(a)–(d)). The anterior-most slice in which the hippocampus is present may, however, be quite dark indicating that grey matter is not yet present (see Figure 16(b)). If this is the case, move one or two slices in a posterior direction until the grey matter (indicated by lighter coloured voxels) within the hippocampus can clearly be seen (see Figure 16(d)). It is at this point that the first slice of the subiculum mask should be created. Another important marker here is the VHS, which gradually becomes clear when moving from anterior to posterior (see Figure 16(d)–(f)). The VHS is an important marker for separating the anterior-most slices of the hippocampus into dorsal (uncul) subiculum and ventral (typical) subiculum portions (see Figure 16(d) and (e)). The VHS may not be readily apparent in the first slice of hippocampus in all subjects.

##### **Step 11**: create the first slice of the subiculum mask

As mentioned above, locate the anterior-most slice of the hippocampus which contains grey matter. Once located, begin creating the first slice of the subiculum mask by placing the pointer in the centre of the lateral-most tip of the ovoid hippocampus (see ‘11’ in the bottom panel of Figure 16(d)). Then trace along the ventral hippocampal wall in a medial direction until reaching the medial-most tip of the hippocampus (see ‘12’ in the bottom panel of Figure 16(d)). Then split the hippocampus into dorsal and ventral portions by drawing a line through the centre of the hippocampus. If the VHS can be seen, use it as a guide to trace through the centre of the hippocampus, following its contour until reaching the starting point. If the VHS cannot be seen, create the line through the approximate centre of the hippocampus being sure to mirror any existing hippocampal contour, until reaching the starting point. Finally, fill in the space enclosed within the newly created boundary.

### From the first slice of the subiculum mask to the tail of the hippocampus

The next step is to repeat the process described in Step 11 for each subsequent slice in a posterior direction. However, as we move posteriorly, anatomical changes occur along the anterior–posterior axis of the hippocampus which result in changes in the tracing method.

#### Histology

An important change in anatomy relates to the VHS and the emergence of the uncul sulcus. Starting at the first slice of the hippocampus and moving in a posterior direction, the hippocampus fattens and, as this occurs, the medial extent of the VHS (see black line in Figure 4(e)) progressively expands until the medial portion of the hippocampus splits into dorsal and ventral components which are separated by the uncul sulcus (see Figure 5(b)). The dorsal wall of the uncul sulcus is occupied by the uncus and is discussed later, in Part 6. The ventral wall is occupied by the subicular cortices comprising the prosubiculum/subiculum and the pre/parasubiculum (see Figure 5(e)).

Between the first slice of the subiculum mask and the final slice of the uncus, the medial and lateral borders of the subiculum undergo change due to the emergence of the CA1 laterally and the pre/parasubiculum medially. According to Ding and Van Hoesen (2015), both CA1 and pre/parasubiculum emerge at the approximate point that the lateral portion of the hippocampus begins to bend in a dorsal direction (see Figure 3(e)). CA1 emerges at the lateral edge of the subiculum and fills the lateral portion of the hippocampus (see Figure 3(e)). The pre/parasubiculum emerges on the medial side of the subiculum (see Figure 3(e)). Therefore, the point at which the lateral portion of the hippocampus begins to bend in a dorsal direction is an important anatomical landmark for adjusting both the medial and lateral borders of the subiculum.

The boundary between the CA1 and subiculum was discussed in Part 3: the CA1 mask. The boundary between the subiculum and the presubiculum is difficult to see on histologically stained tissue, but the approximate point of transition is indicated by the presence of the presubicular ‘islands’ or ‘clouds’ (Green and Mesulam, 1988) (see ‘*’ in Figures 4(b)–12(b)). Unfortunately, these small clusters of cells cannot be seen on T2-weighted images at this resolution. Another indication of the approximate point of transition between these regions can be seen on tissue stained to visualise white matter. Here, the grey matter of the pre/parasubiculum is noticeably darker when compared with the grey matter of the adjacent subiculum (see ″ in Figures 4(c)–14(c)). This intensity change occurs as the transition area of the subiculum and presubiculum is more densely innervated by projections from the perforant pathway, resulting in a darkening of the more white matter rich grey matter in this region (Ding and Van Hoesen, 2015; Iglesias et al., 2015). This intensity change can also be seen on T2-weighted images and, in principle, can be used as a marker to identify the approximate location of the border between the subiculum and pre/parasubiculum. It is important to note that even on histologically stained tissue, this border is difficult to definitively identify. In addition, Ding and Van Hoesen (2015) note that along the anterior–posterior axis of the hippocampus, there is a gradual lateral-to-medial shift in the location of this border. This results in the subiculum gradually becoming more elongated from anterior to posterior. From the crus of the fornix, however, the subiculum may become smaller again as CA1 begins to extend in a medial direction (compare Figures 12(e)–14(e)).

#### Applicability to T2-weighted images

In relation to the anatomical markers mentioned in the previous section, the emergence of the uncul sulcus as the medial portion of the VHS expands and splits the medial hippocampus into ventral and dorsal components can be seen on T2-weighted images. This separation may, however, be difficult to see as the dorsal and ventral portions of the hippocampus press against each other following the emergence of the uncul sulcus (see Figures 5(d)–10(d)). Careful scrutiny of intensity change is crucial, both for identifying the point at which the uncul sulcus emerges and to identify the location of the boundary between the dorsal and ventral portions of the medial hippocampus. The darker voxels of the hippocampal wall can be used as a marker to differentiate between dorsal and ventral components (compare ‘d’ and ‘g’ in Figures 5–10). The point at which the lateral portion of the hippocampus begins to bend in a dorsal direction can also be seen on T2-weighted images.

The lateral border of the subiculum is the ventromedial border of the previously created CA1 mask. In relation to the medial border of the subiculum, as mentioned in the previous section, the intensity change between the subiculum and pre/parasubiculum can, in principle, be used as a marker to identify this border. When looking along the lateral-to-medial extent of the subicular cortices on T2-weighted images, the gradual darkening of the grey matter is clearly observable (see the subicular cortices in Figures 3(a)–13(a)). However, in practice, the gradual intensity change has no clear line of demarcation. Also, the intensity change may not be readily apparent on all slices and may shift in a medial–lateral direction from slice to slice along the longitudinal axis of the hippocampus. Therefore, using this as a marker can result in a disjointed boundary from slice to slice along the axis of the hippocampus. However, to our knowledge, there are no other reliable macroanatomical markers for delineating this boundary on T2-weighted images at this resolution. We describe a method for using this intensity change as a marker of the boundary between the subiculum and pre/parasubiculum in Step 12.

The emergence of the CA1 and pre/parasubiculum must be taken into account and incorporated into the method of tracing the subiculum between the first slice of the mask (created in Step 11) and the tail of the hippocampus. We now describe a method for tracing the subiculum mask between these points, taking these anatomical changes into account.

##### **Step 12**: trace from the first slice of the subiculum mask to the tail of the hippocampus

From the first slice of the subiculum mask, the method described in Step 11 is maintained for each subsequent slice until the point at which the lateral portion of the hippocampus begins to bend in a dorsal direction. As mentioned earlier in this Part, it is at this approximate point that both CA1 and the pre/parasubiculum emerge. CA1 emerges lateral to the subiculum. The medial border of the previously created CA1 mask serves as the lateral border of the subiculum mask along the axis of the hippocampus. The pre/parasubiculum emerges medial to the subiculum. The border of the subiculum and pre/parasubiculum is difficult to see on T2-weighted images. We suggest using the signal intensity change described in the previous section as a marker for this boundary. This may be one of the more difficult borders to identify; however, we are unaware of reliable alternative anatomical markers which can be seen on T2-weighted images at this resolution. We recommend creating the medial boundary of the subiculum at the point where the signal intensity becomes darkest and remains consistently dark over adjacent voxels in a medial direction (see ″ in Figures 4(d)–15(d)). This may be easier to see at a lower magnification and may not always be apparent on every slice.

Once this point is located, to trace the subiculum border in anterior slices (before the appearance of the uncul sulcus), place the pointer on the VHS directly above the darker voxels of the transition area (see ‘4’ in Figure 3(g)) and draw a straight line in a ventromedial direction until reaching the ventral wall of the hippocampus (see ‘5’ in Figure 3(g)). Then trace along the ventral wall in a lateral direction until reaching the ventromedial extent of the CA1 mask. From here, trace along the CA1/subiculum border (the blue line in Figure 3(g)) in a dorsal direction until reaching the VHS. Then trace along the VHS in a medial direction until reaching the starting point. Finally, fill in the space enclosed within the newly created boundary.

Continue this method until the point that the uncul sulcus appears. From here, the subiculum lies along the ventral wall of the uncul sulcus (see Figure 5(g)). Begin tracing the subiculum border by placing the pointer on the dorsal edge of the grey matter above the darker voxels of the transition area (see ‘4’ in Figure 5(g)) and draw a straight line in a ventral direction until reaching the approximate point of the grey–white matter junction (see ‘5’ in Figure 5(g)). This may be difficult to see because the darker colour of the grey matter here can be difficult to differentiate from the white matter. However, the approximate location can be judged by comparing the grey–white matter junction in adjacent regions where the junction can more easily be seen. After reaching the grey–white matter junction, trace along the junction in a lateral direction until reaching the ventromedial extent of the CA1 mask. From here, trace along the CA1–subiculum border in a dorsal direction until reaching the VHS. Then trace along the VHS in a medial direction and continue tracing along the dorsal wall of the subiculum until reaching the starting point. Finally, fill in the space enclosed within the newly created boundary.

This method is maintained along the body of the hippocampus. The only change to mention is that the location of the pre/parasubiculum shifts in a medial direction along the anterior–posterior axis of the hippocampus. This results in a gradual elongation of the subiculum from anterior to posterior. It is important to note this when deciding the medial boundary of the subiculum.

As mentioned earlier in this Part, while the darker voxels are the only consistent anatomical marker for creating the medial border of the subiculum, relying solely on the observable intensity changes can lead to a disjointed border from slice to slice. This method may not perfectly reflect the underlying neuroanatomy. While acknowledging the imperfect nature of this boundary, we recommend, for each slice, that the border is created in reference to the location of the border created in the slices immediately anterior and posterior to it. Observing the location of the darker voxels across two or three slices and creating a ‘mean’ location for the border across each of these slices may result in a smoother border along the anterior–posterior axis.

From the crus of the fornix, continue creating the mask as described above. The lateral border of the subiculum is maintained at the medial border of CA1 as it moves in a medial direction. Moving further posteriorly, this continues to be the case as CA1 extends to encompass the entire space below the DG/CA4 mask (see Figure 14(g)). Continue tracing until the point that the crus of the fornix disappears to become the dorsal wall of the ovoid hippocampus.

From this point, the method described above is continued but the dorsal wall of the subiculum may become more difficult to see as it presses against the dorsal portion of the hippocampus (see Figure 14(d) and (g)). Careful scrutiny is required to differentiate the subiculum from the dorsal portion of the hippocampus. Continue tracing the subiculum either until it becomes impossible to differentiate the dorsal wall of the subiculum or until the final slice of the DG/CA4 mask.

## Part 5: the pre/parasubiculum mask

The presubiculum and parasubiculum are located medial to the subiculum (see Figure 1(d)). The boundary between the pre- and parasubiculum cannot be reliably delineated on MRI at this resolution. We therefore suggest grouping these regions together.

### First slice of the pre/parasubiculum mask

#### Histology

In the serial sections of histologically stained hippocampal tissue analysed by Ding and Van Hoesen (2015), the anterior-most point of the pre/parasubiculum gradually emerges from the medial extent of the subiculum. As with CA1, the gradual emergence of the pre/parasubiculum coincides with the point at which the lateral external digitation of the hippocampus begins to bend in a dorsal direction. Therefore, the slice in which to delineate the first slice of the pre/parasubiculum mask is the first slice in which the lateral-most portion of the hippocampus begins to bend in a dorsal direction.

#### Applicability to T2-weighted images

The point at which the lateral external digitation of the hippocampus first begins to bend in a dorsal direction can be seen on T2-weighted images (see ‘!’ in Figure 3(d)).

##### **Step 13**: create the first slice of the pre/parasubiculum mask

The CA1 and subiculum masks should be created before beginning this step. To create the first slice of the pre/parasubiculum mask, scroll to the slice containing the anterior-most slice of the CA1 mask. The pre/parasubiculum mask will be created medial to the subiculum mask in this slice (see the brown region in Figure 3(g)). Begin by placing the pointer on the dorsomedial extent of the subiculum border (see ‘4’ in Figure 3(g)). Trace in a ventral direction until reaching the ventral extent of the border (see ‘5’ in Figure 3(g)). Then trace in a medial direction along the grey–white matter boundary. We recommend continuing to trace in a medial direction until reaching the end of the line of dark voxels which indicates the end of the medial-most extent of the white matter (see ‘6’ in Figure 3(d) and (g)). When reaching this point, if the VHS is present in the slice, draw a straight line in a dorsal direction until reaching the VHS. If the VHS is not present, draw a straight line in a dorsal direction until reaching a point roughly midway into the hippocampus. Then trace along the VHS or mid-point of the hippocampus in a lateral direction being sure to follow the contour of the hippocampus until reaching the starting point at the dorsomedial border of the subiculum mask (see Figure 3(g)). Finally, fill in the space enclosed within the newly created boundary.

### From the first slice of the pre/parasubiculum mask to the tail of the hippocampus

The next step is to repeat the process described in Step 13 for each subsequent slice in a posterior direction taking anatomical changes along the anterior–posterior axis of the hippocampus into account.

#### Histology

In the anterior-most slice in which the pre/parasubiculum is present, it occupies the ventromedial portion of the thin ribbon of hippocampus (see Figure 3(e)). As described in Part 4: the subiculum, when moving in a posterior direction, the medial portion of the hippocampus splits into dorsal and ventral components, separated by the uncul sulcus. From the point that this split occurs, the pre/parasubiculum occupies the medial-most portion of the ventral wall of the uncul sulcus (see Figure 5(e)).

The lateral border of the pre/parasubiculum is shared with the subiculum along the longitudinal axis of the hippocampus. The anatomical markers for this border are described in Part 4: the subiculum. The medial border is shared with the entorhinal cortex (see Figure 5(e)). In the anterior-most slice that the pre/parasubiculum is present, the entorhinal cortex extends along the entire medial edge of the MTL but is not contiguous with the pre/parasubiculum (see Figure 3(e)). After the uncul sulcus appears, however, the entorhinal cortex lies along the ventromedial edge of the MTL (see Figure 5(e)) and shares a contiguous border with the pre/parasubiculum. According to Ding and Van Hoesen (2015), the junction of the pre/parasubiculum and entorhinal cortex occurs at roughly the point at which the medial-most extent of the subicular cortices turns in a ventral direction (see ‘:’ in Figures 5(e)–11(e)). Therefore, the medial-most point of the grey matter as it bends in a ventral direction is a useful anatomical marker for the boundary between pre/parasubiculum and entorhinal cortex. This marker has previously been used to delineate this border (Wisse et al., 2012; Zeidman et al., 2015). While the pre/parasubiculum continues to occupy the medial-most aspect of the subicular cortices along the axis of the hippocampus (see Figures 5(e)–12(e)), it is important to note that the border of the subiculum and pre/parasubiculum gradually shifts in a medial direction along the anterior–posterior axis. The pre/parasubiculum maintains this position moving into the posterior hippocampus and has been noted to extend further posteriorly until reaching the isthmus, which is occupied by ventral anterior portions of the retrosplenial cortex (Iglesias et al., 2015; http://atlas.brain-map.org). Indeed, the presubiculum may merge and transition with the retrosplenial cortex in this region (Berger et al., 1997).

#### Applicability to T2-weighted images

Each of the landmarks described above can be seen on T2-weighted images. As described in Part 4: the subiculum, the uncul sulcus emerges as the medial portion of the VHS expands and splits the medial hippocampus into ventral and dorsal components.

In relation to the lateral and medial borders of the pre/parasubiculum, the lateral border is shared with the medial border of the subiculum mask which has been created in a previous step. The medial border lies at the medial-most point of the subicular cortices at the approximate point where the ventromedial portion of hippocampus bends sharply in a ventral direction. This can be seen on T2-weighted images (see ‘:’ in Figures 5(g)–14(g)).

##### **Step 14**: trace from the first slice of the pre/parasubiculum mask to the tail of the hippocampus

For each slice immediately posterior to the first slice of the pre/parasubiculum mask, the method described in Step 13 is maintained until the point that the uncul sulcus appears. After this point, it is important that the pre/parasubiculum mask is maintained on the ventral wall of the uncul sulcus, medial to the subiculum mask.

To trace the pre/parasubiculum mask from the point that the uncul sulcus is present, begin by placing the pointer on the dorsal-most point of the medial subiculum border (see ‘4’ in Figure 5(g)). From here, trace a line along the ventral wall of the uncul sulcus, indicated by the darker line of voxels (see and compare Figure 5(d) and (g)) in a medial direction until reaching the medial-most point of the grey matter (see ‘:’ in Figure 5(g)). The ventral wall of the uncul sulcus may be difficult to identify. Careful scrutiny of the intensity change is required here. From here, draw a straight line in a ventrolateral direction until reaching the medial-most part of the grey–white matter junction (see ‘6’ in Figure 5(g)). Then draw a line along the grey–white matter boundary in a lateral direction until reaching the ventromedial-most point of the subiculum border. Trace along the medial border of the subiculum in a dorsal direction until reaching the starting point. Finally, fill in the space encompassed by this boundary. This method is maintained along the body of the hippocampus.

When entering the tail of the hippocampus, continue creating the mask as described above. Maintain the lateral border of the pre/parasubiculum mask at the medial border of the subiculum mask and the medial border at the point of the medial-most extent of the grey matter. This results in the mask gradually becoming smaller when moving posteriorly. The pre/parasubiculum transitions to the retrosplenial cortex at the approximate location of the isthmus, which is located medial to the posterior-most portion of the hippocampus (see Figures 15(g) and 24, and see http://atlas.brain-map.org). Therefore, the posterior portion of this mask may incorporate a small amount of the retrosplenial cortex. To keep the mask as ‘pure’ as possible, create the final slice of the pre/parasubiculum mask on the same slice as the final slice of the DG/CA4 mask.

## Part 6: the uncus mask

The neuroanatomy of the uncus is complex. The uncus contains each of the subregions found in the ‘typical’ hippocampus (DG, CA4-1, subiculum, pre/parasubiculum). Importantly, while ‘uncul’ subregions share many features with ‘typical’ subregions, modifications in cyto- and chemo-architecture are observed between ‘uncul’ and ‘typical’ subregions (Ding and Van Hoesen, 2015; Zeidman and Maguire, 2016). This has led Ding and Van Hoesen to differentiate between ‘uncul’ and ‘typical’ subregions. Ding and Van Hoesen’s recent anatomical investigations are, arguably, the most detailed investigations of anterior hippocampal anatomy to date and are an invaluable resource for more accurate delineation of the uncul subregions. Unfortunately, it is impossible to differentiate between the substructures of the uncus at the resolution of 3T MRI scans. However, the delineations described by Ding and Van Hoesen (2015) provide important insights into the extent to which the uncul subregions occupy the anterior hippocampus. Here, we apply these insights to the creation of a single mask of the uncus, incorporating all its subregions. Importantly, our uncus mask extends more anteriorly than those adopted in previous hippocampal subregion segmentations (Zeidman et al., 2015), a decision that we believe is warranted in light of the recent anatomical evidence.

### First slice of the uncus mask

#### Histology

Subfields of the uncus are present in the anterior-most portions of the hippocampus. As described in Part 4: the subiculum, Ding and Van Hoesen (2015) suggest that while the anterior-most portion of the hippocampus contains subiculum, the ventral portion contains ‘typical’ subiculum (created in a previous step) and the dorsal portion contains ‘uncul’ subiculum (relevant to this step) which, when moving posteriorly, becomes part of the uncus. We, therefore, recommend creating the first slice of the uncus mask in the dorsal portion of the first slice of the hippocampus.

#### Applicability to T2-weighted images

The anterior-most slice of the hippocampus can be seen on T2-weighted images and is described in Part 4: the subiculum.

##### **Step 15**: trace the first slice of uncus mask

To create the first slice of the uncus mask, locate the anterior-most slice of the subiculum mask. The first slice of the uncus mask will be created on the same slice. Begin tracing the uncus mask by placing the pointer in the centre of the lateral-most tip of the ovoid hippocampus (see ‘11’ in the bottom panel of Figure 16(d)). Then trace along the dorsal wall of the hippocampus in a medial direction until reaching the medial-most tip of the hippocampus (see ‘12’ in the bottom panel of Figure 16(d)). From there, trace along the dorsal border of the subiculum mask in a ventrolateral direction until reaching the starting point. Finally, fill in the space enclosed within the newly created boundary.

### From the first slice of the uncus mask until the final slice of the uncus

The next step is to repeat the process described in Step 15 for each subsequent slice in a posterior direction. However, as we move posteriorly, anatomical changes occur along the anterior–posterior axis of the uncus which result in changes in the tracing method.

#### Histology

A detailed summary of uncul subfield anatomy is beyond the scope of this article. While we are aware that the ‘uncul’ subregions transition along the anterior–posterior uncul axis in a similar manner to the ‘typical’ subfields along the hippocampal axis, here, we refer to the uncus as a unitary structure and not to its specific subregions.

In the anterior-most slices, the uncus occupies the entire dorsal portion of the thin ribbon of hippocampus (Ding and Van Hoesen, 2015) (see the lower panels of Figure 16(d) and (e)). This remains the case until the emergence of CA1. As described in Part 3: the CA1 mask, CA1 emerges at roughly the point that the lateral portion of the hippocampus begins to bend in a dorsal direction (see Figures 3(e) and 16(f)). The lateral border of the uncus shifts medially with the emergence of the CA1 (see Figures 3(e) and 16(f)). Therefore, the point at which the lateral portion of the hippocampus begins to bend in a dorsal direction is an important landmark for adjusting the lateral border of the uncus.

The boundary between the ‘typical’ CA1 and the uncus is difficult to see on histologically stained tissue. However, Ding and Van Hoesen (2015) provide cyto- and chemo-architectural evidence that the transition occurs at the approximate point of the depression at the medial extent of the lateral external digitation (see ‘!’ in Figures 3(b)–10(b)). This is another important marker for the lateral border of the uncus mask. Moving posteriorly, as the lateral portion of the hippocampus becomes fatter, this depression along with the lateral border of the uncus recedes in a medial direction (see medial progression of ‘!’ from Figures 3(b)–10(b)).

In parallel, when moving in a posterior direction, the medial portion of the hippocampus splits into dorsal and ventral components, separated by the uncul sulcus. As described in Part 4: the subiculum, from the point that this split occurs, the uncus occupies the dorsal wall of the uncul sulcus (see Figure 5(e)). The uncul sulcus is, therefore, another important landmark for delineating the uncus.

At approximately the same point that the uncul sulcus appears, the dorsomedial portion of the uncus bends in a dorsal direction (see the aquamarine line in Figure 5(b)). This dorsomedial portion of the uncus appears as a strip of tissue connecting the hippocampus with the amygdala (see the aquamarine line in Figures 5(b)–6(b)). This portion of the uncus is oriented in a dorsal–ventral direction and contains subregions which Ding and Van Hoesen (2015) refer to as the ‘vertical’ subfields. These ‘vertical’ subfields extend dorsally and transition with the hippocampo-amygdalar transition area (HATA) which lies in the dorsal portion of this vertically oriented strip of tissue (see ‘b’ in Figures 5 and 6). Importantly, according to Ding and Van Hoesen (2015), the transition between the vertical subfields and the HATA occurs at the approximate point that the dorsomedial wall of the hippocampus bends in a dorsal direction. In other words, at the point the dorsomedial wall of the hippocampus meets the ventrolateral wall of the vertically oriented tissue containing the vertical subfields and HATA (see ‘7’ in Figures 5(b)–6(b)). This is an important anatomical marker for delineating the medial extent of the uncus mask. Moving posteriorly, the HATA recedes in a dorsal direction, eventually disappearing until the dorsomedial portion of the uncus contains only the vertical subfields.

The depression at the medial extent of the lateral external digitation remains an important marker for the lateral border of the uncus until the point that the dorsolateral extent of the uncul sulcus completes its dorsal extension and splits the uncus from the rest of the hippocampus (described in Part 1: the DG/CA4 mask). Following the separation of the uncus from the rest of the hippocampus, it can be seen as a solitary island floating medial to the hippocampus ‘proper’ (see Figures 10(e) and 11(e)).

#### Applicability to T2-weighted images

In relation to the markers which are useful for delineating the boundaries of the uncus described above, the point at which the lateral portion of the hippocampus begins to bend in a dorsal direction can be seen on T2-weighted images and is described in Part 3: the CA1 mask (see Figure 3(d)). Likewise, the depression at the medial extent of the lateral external digitation can be seen on T2 (see ‘!’ in Figures 3(d)–10(d)) and is described in Part 2: the CA3/2 mask.

The uncul sulcus is easy to see on histologically stained tissue. However, in vivo, the dorsal and ventral walls of the uncul sulcus remain tightly pressed against each other, making identification on T2-weighed scans difficult. It can, however, generally be made out as a line of darker voxels separating the ventral and dorsal walls (see Figures 7– 10(d) and compare with the outline presented in 7–10(g)). Moving posteriorly, the separation of the uncus from the hippocampus proper, as the lateral extent of the uncul sulcus extends in a dorsal direction, can be seen and is described in Part 1: the DG/CA4 mask.

The point at which the dorsomedial wall of the hippocampus meets the ventrolateral wall of the vertically oriented tissue containing the vertical subfields and HATA can also easily be seen on T2-weighted images (see ‘7’ in Figures 5(d)–8(d)).

The remaining ‘island’ of the posterior uncus can also be difficult to see on T2-weighted images. Careful scrutiny of the signal intensity is required. As a general rule, moving posteriorly, the uncus can be identified as an increasingly small collection of grey voxels, lying medial to the hippocampus proper. Here, the grey voxels of the uncus contrast with the surrounding white and black voxels of the CSF and blood vessels, respectively (see and compare the uncus in Figure 11(d) and (g)).

##### **Step 16**: trace from the first slice of the uncus mask to the final slice of the uncus

Repeat the process described in Step 15 for each subsequent slice in a posterior direction. In anterior slices, the hippocampus is surrounded by white matter such that the medial and lateral boundaries are quite clear (see Figure 16(c)–(e)). Moving posteriorly, however, the medial side of the hippocampus ‘opens’ making medial border delineation tricky (see Figures 3(d) - 6(d). As the medial hippocampus opens, a line of white matter can be seen extending medially along the dorsal wall of the hippocampus (see ‘7’ in Figures 3(d)–6(d)). We recommend using the medial-most end of this line of white matter for delineating the medial border of the uncus.

From the point that the medial hippocampus begins to open, begin tracing the uncus mask by placing the pointer on the lateral-most edge of the hippocampus and tracing along the dorsal wall of the hippocampus in a medial direction until reaching the medial-most extent of the white matter (see ‘7’ in the bottom panel of Figure 16(e)). From here, draw in a ventrolateral direction to the dorsomedial corner of the subiculum mask. Then trace along the dorsal border of the subiculum mask in a lateral direction until reaching the start point. Fill the boundary created in this step.

Continue this method until the point that the CA1 mask appears. From here, begin tracing by placing the pointer on the dorsomedial corner of the CA1 mask and trace along the dorsal wall of the hippocampus in a medial direction until reaching the medial-most extent of the white matter (see ‘7’ in Figure 3(g)). From here, draw in a ventrolateral direction to the dorsomedial corner of the pre/parasubiculum mask. Then trace along the dorsal border of the pre/parasubiculum and subiculum mask in a lateral direction until reaching the medial border of the CA1 mask. From here, draw along the CA1 border in a dorsal direction until reaching the start point. Then fill the boundary created in this step.

Moving posteriorly, continue this method until the uncul sulcus appears (see Figure 5(g)). From this point, begin tracing by placing the pointer on the dorsomedial corner of the CA1 mask (this should now coincide with the depression of the medial extent of the lateral external digitation) and trace along the dorsal wall of the hippocampus in a medial direction until reaching the medial-most extent of the white matter (see ‘7’ in Figure 5(g)). From here, draw a straight line in a medial direction to the medial wall of the uncus (see ‘8’ in Figure 5(g)). Then trace along the dorsal wall of the uncul sulcus in a lateral direction until reaching the lateral-most extent of the uncul sulcus. As described in Part 1: the DG/CA4 mask, this may be apparent as a collection of brighter voxels indicating the presence of CSF. From here, draw a straight line in a dorsal direction to the starting point at the depression of the medial extent of the lateral external digitation. This line may be vertical or oblique depending on the neuroanatomy of the individual. Then fill the boundary created in this step.

This method should be continued until the point that the uncus splits from the lateral hippocampus. Two important changes are, however, necessary when moving posteriorly. The first relates to the medial extent of the uncus mask. As mentioned earlier in this Part, the medial-most extent of the uncus gradually turns in a dorsal direction. This medial portion of the uncus contains the vertical subfields and, more dorsally, the HATA. From the point that the medial portion of the uncus begins to bend in a dorsal direction, we recommend continuing to create the medial border of the uncus by drawing a straight line from the point that the dorsomedial wall of the hippocampus meets the ventrolateral wall of the vertically oriented tissue (see ‘7’ in Figures 5(g)–8(g)) to the medial wall of the uncus (see ‘8’ in Figures 5(g)–8(g)). We have chosen this method as the work by Ding and Van Hoesen (2015) suggests that the HATA does not extend below this point. Therefore, using this point as a marker should result in little contamination from HATA in the uncus mask. The second important change relates to the lateral border of the uncus mask. Continue creating this border by drawing a straight line between the depression of the medial extent of the lateral external digitation and the lateral-most extent of the uncul sulcus. Importantly, as described in Part 1: the DG/CA4 mask, the lateral extent of the uncul sulcus extends dorsally until the uncus is separated from the hippocampus proper. During this progression, be sure to maintain the lateral border of the uncus mask from the ventral-most point of the depression of the medial extent of the lateral external digitation to the dorsolateral-most point of the uncul sulcus until the point that the uncus splits from the hippocampus proper (see the progression from Figures 5(g)–10(g)).

As described earlier in this Part, following the separation of the posterior uncus from the rest of the hippocampus, the uncus can be seen as a solitary island floating medial to the hippocampus ‘proper’ (see Figures 10(e) and 11(e)). To create the uncul boundary from this point, simply trace around the island of remnant tissue (see Figures 10(g) and 11(g)). This may be difficult to see and so careful scrutiny of signal intensity is necessary here. This method results in a mask which encompasses all the uncul subregions.

**Figure 22:**
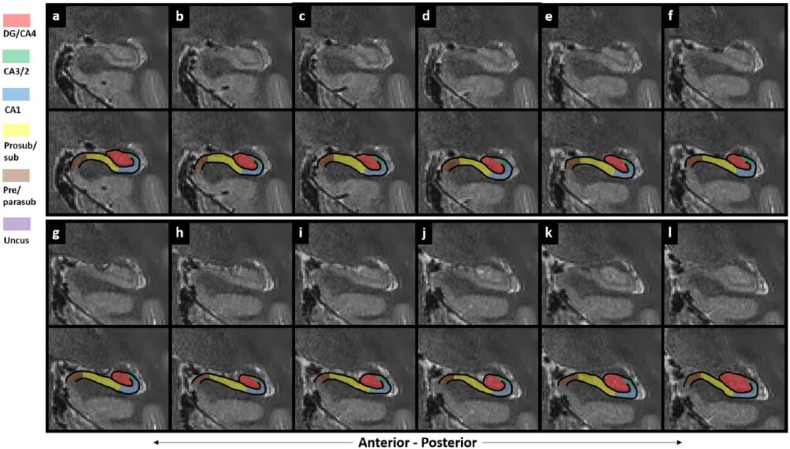


**Figure 23:**
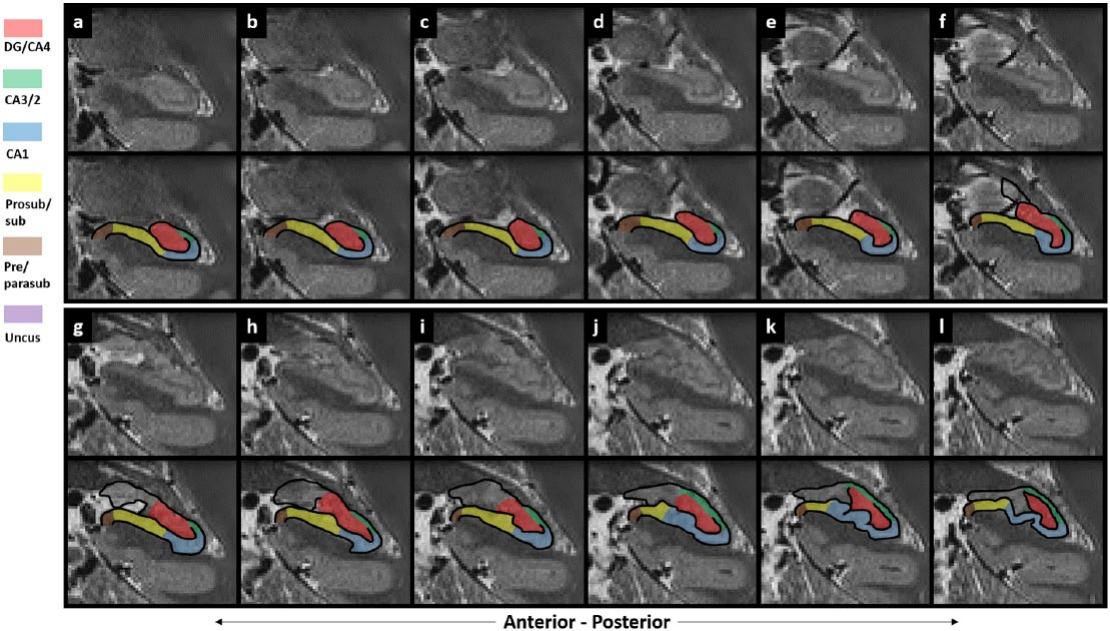


**Figure 24:**
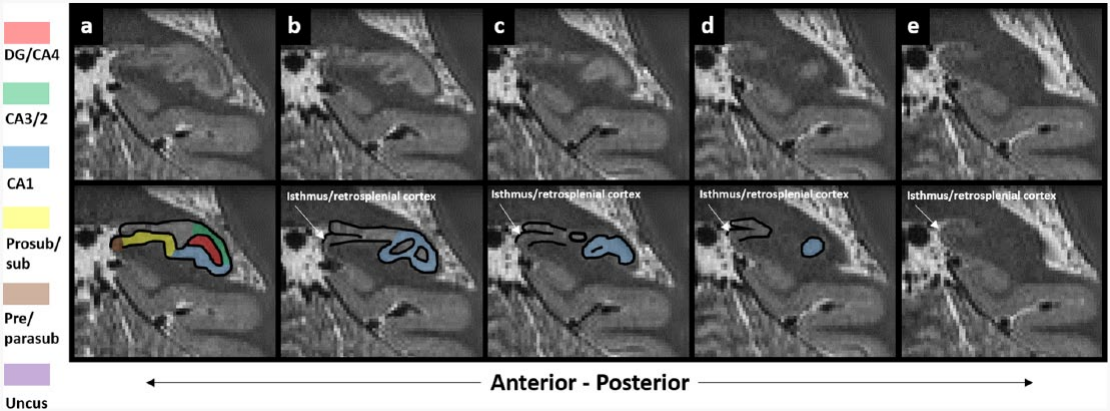


This concludes the segmentation protocol. Note that an example, slice-by-slice, sequence of images spanning the longitudinal axis of the hippocampus in its entirety, overlaid with our subregion delineations are shown in Figures 18–24.

Two researchers (one experienced and one complete novice) independently segmented six hippocampi according to the protocol. Reliability analyses for each subfield were conducted using the Dice overlap metric (Dice, 1945) to produce a score between 0 (no overlap) and 1 (perfect overlap). Inter-rater reliability was 0.83 for DG/CA4, 0.67 for CA3/2, 0.77 for CA1, 0.77 for subiculum, 0.68 for pre/parasubiculum and 0.81 for the uncus. These values are equivalent to those reported in the extant literature (Bonnici et al., 2012; Palombo et al., 2013). Intra-rater reliability was measured 3 months apart and showed high concordance between the segmentations at the two different time points: 0.86 for DG/CA4, 0.76 for CA3/2, 0.85 for CA1, 0.86 for subiculum, 0.75 for pre/parasubiculum and 0.87 for the uncus.

## Discussion

We described a detailed, step-by-step guide to the manual segmentation of human hippocampal subregions on high-resolution T2-weighted MRIs acquired on a 3T scanner. In this protocol, we delineate six subregions – the DG/CA4, CA3/2, CA1, subiculum, pre/parasubiculum and the uncus along the entire anterior–posterior axis of the hippocampus. To define the borders of each subregion, we combine knowledge from previously published hippocampal segmentation protocols and recent histological investigations of human hippocampal neuroanatomy.

A consensus regarding the location of hippocampal subregion boundaries on MRI scans is yet to be reached. Consequently, methodologies for border delineation vary greatly between research groups (Yushkevich et al., 2015). This is hardly surprising because even at the microscopic level, definitive subregion delineation can be difficult as there are no abrupt borders between subregions, rather they transition gradually. As hippocampal subregion segmentation is an evolving field of research, we do not suggest that the protocol outlined here is a definitive or final method. Rather, until wide agreement has been achieved, we believe this protocol is faithful to the current understanding of hippocampal neuroanatomy and is novel in providing, for the first time, a ‘tutorial’-style guide which can be followed by experts and non-experts alike. Combined with the detailed figures comparing histology to MRI, we hope that this method can be utilised by researchers to acquire the familiarity and skills necessary to identify important macroanatomical landmarks with which to determine the likely location of subregion boundaries on MRI.

Our protocol contains a number of differences compared to the extant literature. Considering first the anterior hippocampus, we draw heavily on the recent investigations of Ding and Van Hoesen (2015; see also Zeidman et al., 2015; Zeidman and Maguire, 2016) and include the uncus as a separate mask. Few previous studies separately delineated the uncus, instead incorporating both ‘typical’ and uncul subregions into one mask (Adler et al., 2014; Iglesias et al., 2015; Kulaga-Yoskovitz et al., 2015; Wood et al., 2015). However, uncul subregions have cellular ‘peculiarities’ (McLardy, 1963; Zeidman and Maguire, 2016) and unique patterns of connectivity (Insausti and Munoz, 2001; Rosene and van Hoesen, 1987). We therefore think it is important to include the uncus as a separate mask in a hippocampal segmentation protocol. We acknowledge that the uncus is a complex structure and should ideally be subdivided into its subregions. However, they cannot be reliably differentiated at the resolution of the scans used here and so we incorporate all the uncul subregions into one mask.

Another distinctive element of our protocol is the separation of the subiculum and pre/parasubiculum. Few previous studies have taken this approach (Iglesias et al., 2015; Zeidman et al., 2015) with most methods incorporating the pre/parasubiculum and subiculum into a single mask (Adler et al., 2014; Kulaga-Yoskovitz et al., 2015; Winterburn et al., 2013). However, the human subiculum and pre/parasubiculum have different patterns of functional connectivity (Maass et al., 2015) and also differentially contribute to specific cognitive processes (Maass et al., 2014; Zeidman et al., 2015). While incorporating the entire subicular complex into one mask may be expedient, this approach limits the ability to detect functional differences between its medial (pre/parasubiculum) and lateral (prosubiculum/subiculum) portions. Accordingly, we strongly believe that these regions should be separately segmented in any hippocampal segmentation protocol.

An additional distinguishing feature of our method concerns the location of the border between the CA1 and subiculum masks. As observed by Yushkevich et al. (2015), the placement of this boundary shows the greatest disagreement among extant segmentation protocols. While some groups extend CA1 medially, creating the boundary in alignment with the medial-most edge of the DG (Adler et al., 2014; Bonnici et al., 2012; Wisse et al., 2012; Wood et al., 2015), others create this border in a more lateral location (Iglesias et al., 2015; Kulaga-Yoskovitz et al., 2015; Malykhin et al., 2010). In microscopy studies, the CA1–subiculum boundary is generally considered to lie in a more lateral location and may not extend past the mid-point of the DG until more posterior portions of the hippocampus (Ding and Van Hoesen, 2015; Duvernoy et al., 2013). However, there is also likely to be a degree of individual variability in the location of this boundary. We suggest that the CA1 mask is commonly overextended in a medial direction and likely incorporates a large portion of the subiculum in many studies. While the exact location of this boundary is difficult to identify as it moves medially along the anterior–posterior axis of the hippocampus, the boundary we utilise here accords with recent histological investigations (Ding and Van Hoesen, 2015). Many studies also incorporate swaths of the uncul subfields in their anterior CA1 delineations (Adler et al., 2014; Iglesias et al., 2015; Wisse et al., 2012). This approach makes it difficult to know whether a significant result attributed to CA1 in these studies is truly driven by typical CA1 or by uncul subfields.

For the body of the hippocampus, we draw heavily on the work of Duvernoy et al. (2013), and with the exception of the differences noted above, our protocol does not differ greatly from recently described methods, but importantly we lay out in detail how to implement this approach.

In contrast, the tail of the hippocampus (i.e. those portions lying posterior to the crus of the fornix) is arguably the most difficult portion of the hippocampus to delineate. To our knowledge, there are no detailed neuroanatomical investigations of the human hippocampal tail with which to accurately guide segmentation of this region. Some groups delineate subregions of the hippocampus up until the crus of the fornix and then create a separate mask encompassing the entire portion of the hippocampus lying posterior to this (Iglesias et al., 2015), while others attempt to delineate subregions within the hippocampal tail (Winterburn et al., 2013; Wisse et al., 2012; Wood et al., 2015). Here, we draw on the delineation decisions made in these prior studies in combination with the observations of Duvernoy et al. (2013). In addition, we have attempted to extend some principles of hippocampal neuroanatomy found in the body of the hippocampus, to the hippocampal tail. This is illustrated in our continuing the CA3/2 region into the tail of the hippocampus and transitioning from CA3/2 to CA1 at the final slice of the DG based on the fanning out of the cellular layer as seen on histologically stained tissue (see Part 2: the CA3/2 mask). Overall, the posterior hippocampus contains the most uncertain part of this protocol. Further neuroanatomical investigations are necessary to more reliably inform future iterations.

**Figure 25.**
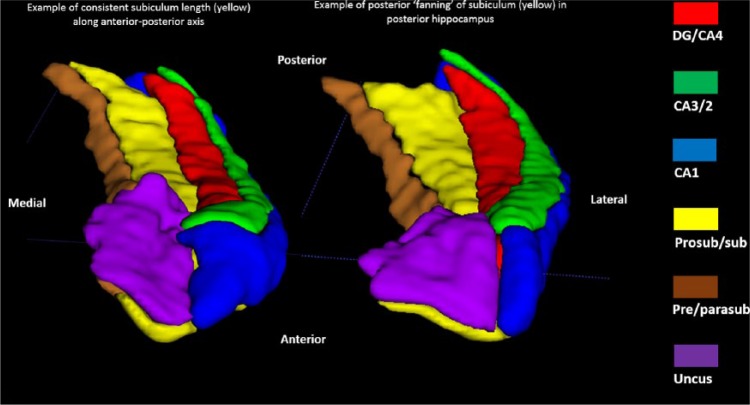
Individual variability in subiculum length. On the left, an example of a consistent subiculum length (in yellow) along the anterior-posterior axis. On the right an example of posterior ‘fanning’ of the subiculum (in yellow).

In addition to being the most difficult portion of the hippocampus to delineate, it is in the tail of the hippocampus that the greatest degree of individual variability is evident on MRI scans. Morphological differences in the hippocampal tail are common. In addition, individual differences in the degree of hippocampal flexure and head positioning in the scanner can result in variability in the angle that coronal slices are taken through the hippocampal tail. These issues combine to render the identification of some landmarks difficult. While individual variability is an important issue, a healthy hippocampus does not typically stray drastically from what is considered a ‘usual’ morphology. However, no hippocampus is identical and even small changes in morphology can make interpretation of MRIs confusing. Variabilities in the length or shape of subregions are common. For example, in some individuals, the subiculum lengthens in posterior regions, appearing to fan out in a medial direction, while in others it remains a consistent length down the long axis of the hippocampus (see Figure 25). A thorough discussion of individual variability in hippocampal morphology is beyond the scope of this article and, to the best of our knowledge, morphological variability of hippocampal subregions has not been systematically investigated in the human brain. This is clearly an important issue that should be addressed directly in future studies.

**Figure 26.**
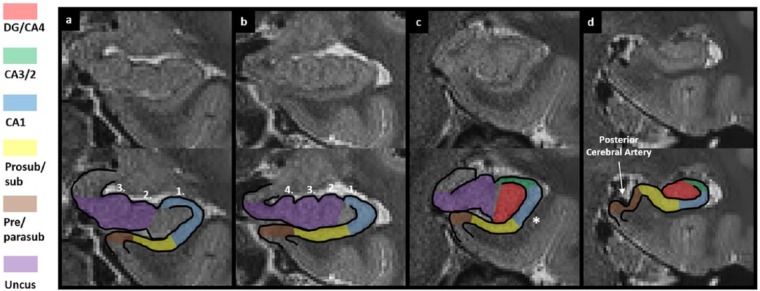
Individual variability in the anterior hippocampus 1. Examples from different individuals showing commonly observed morphological differences in the anterior hippocampus and body of the hippocampus. (a) Anterior hippocampus with three external digitations on the dorsal wall, (b) anterior hippocampus with four external digitations on the dorsal wall and (c) a ‘steep’ lateral wall in the anterior hippocampus (see ‘*’). This can make CA1–subiculum border delineation difficult. (d) Cortical tissue deformation as a result of pressure from adjacent blood vessels.

**Figure 27.**
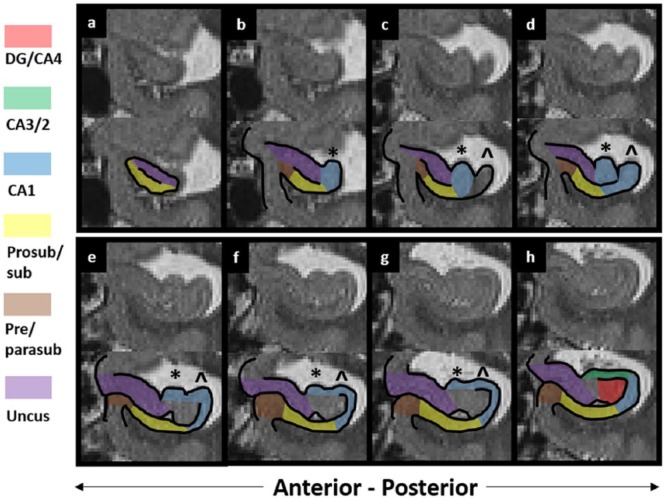
Individual variability in the anterior hippocampus 2. Example of a common morphological characteristic in the anterior hippocampus whereby after initial bending of the lateral external digitation (‘*’ in (b)), another digitation emerges laterally when moving in a posterior direction (‘^’ in 27(c)). This can make delineation of the CA1 region confusing. In these cases, we recommend considering both digitations to contain ‘typical’ CA1 as, when moving posteriorly, they generally merge to create a single digitation which becomes the dorsal wall of the body of the hippocampus (see Figure 27(h)). ‘a’ represents the anterior-most slice, with each subsequent panel (‘b’–‘h’) representing a contiguous slice in the posterior direction.

**Figure 28.**
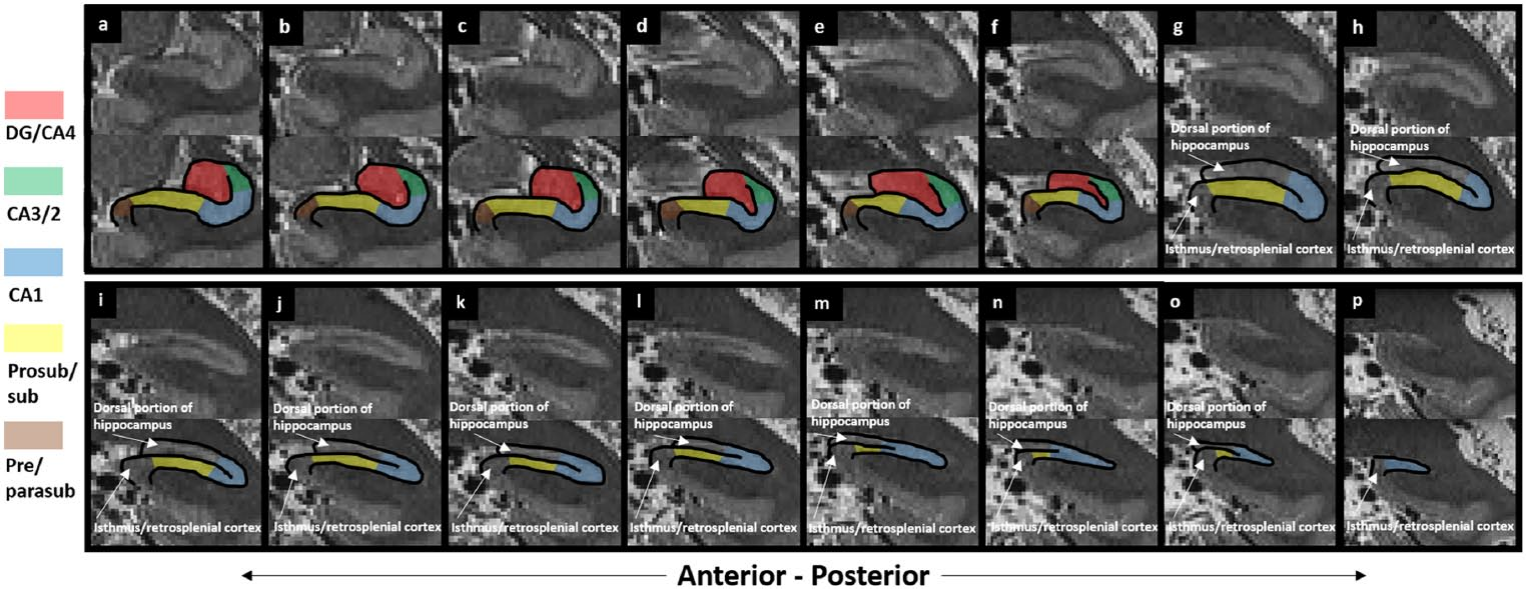
Individual variability in the posterior hippocampus 1. In this common variant of posterior hippocampal morphology, the ‘ovoid’ portion of the posterior hippocampus noted in the protocol does not emerge. Rather, the dorsal and ventral portions of the hippocampus close on each other, making delineation of these regions difficult. Here, we offer an example of how we segment in the context of this particular morphological characteristic. ‘a’ represents the anterior-most slice, with each subsequent panel (‘b’–‘p’) representing a contiguous slice in the posterior direction.

**Figure 29.**
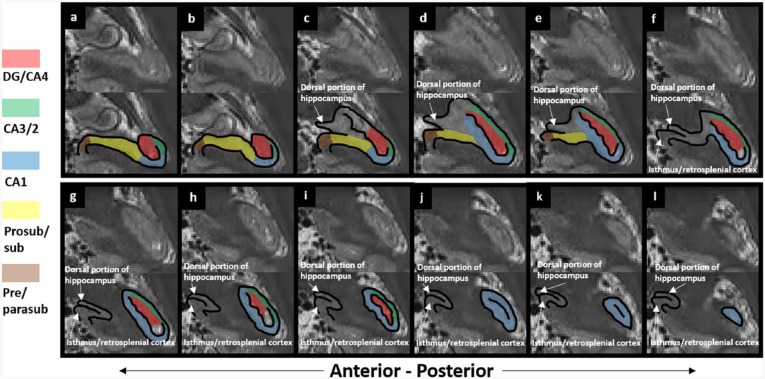
Individual variability in the posterior hippocampus 2. In this common variant of posterior hippocampal morphology, the ‘ovoid’ portion of the posterior hippocampus separates to become an island in a comparatively anterior portion of the hippocampus when compared with that noted in our protocol. Here, we offer an example of how we segment in the context of this particular morphological characteristic. ‘a’ represents the anterior-most slice, with each subsequent panel (‘b’–‘l’) representing a contiguous slice in the posterior direction.

Taking these individual differences into account, we acknowledge that it is unfeasible to lay out a single protocol that can perfectly encapsulate all hippocampi. However, while it may be necessary to make amendments to elements of this protocol for ‘unusual’ hippocampi, we believe its core remains sound and its principles can be extended to less common hippocampal morphologies on a case-by-case basis. Figures 26–29 show some examples of commonly observed morphological characteristics which differ from elements described in this protocol. We have overlaid these images with examples of how we do the delineations in these cases. It is important to reiterate that delineations of the posterior hippocampus are the most uncertain element of this protocol and will be the most likely to undergo future amendments as more detailed investigations of the human posterior hippocampus are conducted.

For the reasons noted above, although manual segmentation requires more time, we believe it to be superior to automated methods when it comes to accurate delineation of hippocampal subfields, especially in anterior and posterior portions of the hippocampus. Importantly, automated methods are largely based on initial manual delineations. Considering there is yet to be a consensus on accurate methods of manual subregion delineation (Yushkevich et al., 2015), the accuracy of automated techniques which are based on initial manual segmentation is questionable. Automated methods are only as accurate as the data fed into them and likely sacrifice accuracy for speed. Until a field-wide consensus is reached, an aspiration currently being pursued by The Hippocampal Subfield Group (www.hippocampalsubfields.com), manual segmentation remains the gold standard.

It is important to note that in our protocol, there are two regions of tissue that are not included in any mask. The first is in the anterior hippocampus between the uncus, DG, CA3/2 and CA1 masks (see these unmasked areas in ‘e’, ‘f’ and ‘g’ of Figures 5–9). These regions are not included as they likely contain a mix of subregions which cannot be easily separated on the T2-weighted images used here. Even on histologically stained tissue, subregions in these areas can be difficult to delineate (see the complexity of the unmasked tissue in Figures 5(e) and 7(e)). Second, we do not include the dorsal portion of the posterior hippocampus (see unmasked areas in ‘e’, ‘f’ and ‘g’ of Figures 13–15). This region also contains a mixture of DG and CA4-1 which cannot be differentiated at the resolution of our images. Overall, while bearing in mind the already difficult task of accurately encompassing the neuroanatomical substrates of interest within each mask, our decision to leave some tissues unaccounted for reflects the attempt to keep each mask as ‘pure’ as possible by not incorporating these two particularly ‘messy’ areas.

A number of common difficulties occur in the course of delineating hippocampal subregions. One issue relates to the inherent difficulty in identifying borders. When borders for any given subregion are difficult to identify, as mentioned in Step 12, we recommend as a general rule that the border is created in reference to the location of that same border in slices immediately anterior or posterior to the problem slices. This should help maintain consistency across problem slices. Another issue relates to naturally occurring cavities which are commonly found in the VHS. These can be seen as collections of hyperintense bright voxels within the VHS (see Figure 17(a) and (b)). These cavities are hypothesised to be remnants of the hippocampal folding process which occurs during foetal brain development (Sasaki et al., 1993; Van Veluw et al., 2013). Cavities vary in size. Larger cavities bulge against the walls of the VHS and can result in significant deformation of the surrounding cortical tissue. This commonly occurs adjacent to CA1 and the subiculum and can result in spontaneous thinning of these subregions in slices preceding and/or proceeding slices that contain the cavity. Therefore, the thickness of CA1 and the subiculum can significantly fluctuate along the anterior–posterior axis of the hippocampus making delineations based on cortical thickness problematic. It is important, therefore, to maintain the CA1 and subiculum boundaries in line with the protocol, even if the cortices spontaneously thin as a result of VHS cavities.

Another issue arises in relation to the VHS. While it is seen as an inverted ‘C’ of dark voxels along the anterior–posterior axis of the hippocampus (described in Part 1: the DG/CA4 mask), the ventrolateral portion can sometimes spontaneously extend in a ventral direction (see Figure 17(c) and (d)). This can occur in the presence or absence of cavities and, on histology, the ventral extension of the VHS coincides with a thinning of the adjacent cortical areas (see Figure 17(c)). On MRI, the spontaneous ventral extension of the VHS can be confusing and make delineation of the adjacent DG, CA1 and subiculum difficult, not only in the slice in which the extension is clear but also in slices anterior and posterior to the extension. When this occurs, we recommend not including the space within the dark region of the ventrally extended VHS and continuing to trace the subregions in accordance with the protocol relevant to that portion of hippocampus (see Figure 17(f) for an example).

In closing, we intend this detailed protocol to act as a guide to segmenting hippocampal subregions along the entire length of the hippocampus in a clear, step-by-step manner. While this will be useful for anyone with access to a 3T scanner, the method can also be adapted to suit the interests of the individual researcher or clinician. In particular, we hope that newcomers to hippocampal subregion segmentation can use this guide to create a mental template with which to more easily recognise the sometimes ambiguous features of the hippocampus as seen on MRIs. For experts in hippocampal segmentation, our style of presentation is undoubtedly simplistic, but we hope that they too will find points of interest in this method, not least the incorporation of the most recent microscopy data. We do not suggest this protocol is definitive or final. We hope it will contribute to ongoing efforts in the field to create a unified methodology in the future, while in the meantime providing practical assistance to those currently examining hippocampal subregions in humans.

## References

[bibr1-2398212817701448] Adler DH Liu AY Pluta J (2012) Reconstruction of the human hippocampus in 3D from histology and high-resolution ex-vivo MRI. In: Proceedings of the IEEE International Symposium on Biomedical Imaging, Barcelona, 2–5 5, pp. 294–297. New York: IEEE. 10.1109/ISBI.2012.6235542 PMC3892900 24443672

[bibr2-2398212817701448] Adler DH Pluta J Kadivar S (2014) Histology-derived volumetric annotation of the human hippocampal subfields in postmortem MRI. NeuroImage 84: 505–523. 24036353 10.1016/j.neuroimage.2013.08.067 PMC3864597

[bibr3-2398212817701448] Amaral DG Lavanex P (2007) Hippocampal Neuroanatomy: The Hippocampus Book. Oxford: Oxford University Press.

[bibr4-2398212817701448] Berger B Alvarez C Pelaprat D (1997) Retrosplenial/presubicular continuum in primates: A developmental approach in fetal macaques using neurotensin and parvalbumin as markers. Developmental Brain Research 101(1–2): 207–224. 9263594 10.1016/s0165-3806(97)00067-9

[bibr5-2398212817701448] Berron D Schutze H Maass A (2016) Strong evidence for pattern separation in human dentate gyrus. Journal of Neuroscience 36(29): 7569–7579. 27445136 10.1523/JNEUROSCI.0518-16.2016 PMC6705559

[bibr6-2398212817701448] Bonnici HM Chadwick MJ Kumaran D (2012) Multi-voxel pattern analysis in human hippocampal subfields. Frontiers in Human Neuroscience 6: 290. 23087638 10.3389/fnhum.2012.00290 PMC3474998

[bibr7-2398212817701448] Braak H Braak E Yilmazer D (1996) Functional anatomy of human hippocampal formation and related structures. Journal of Child Neurology 11(4): 265–275. 8807415 10.1177/088307389601100402

[bibr8-2398212817701448] Collin SH Milivojevic B Doeller CF (2015) Memory hierarchies map onto the hippocampal long axis in humans. Nature Neuroscience 18(11): 1562–1564. 26479587 10.1038/nn.4138 PMC4665212

[bibr9-2398212817701448] Coras R Pauli E Li J (2014) Differential influence of hippocampal subfields to memory formation: Insights from patients with temporal lobe epilepsy. Brain 137(Pt 7): 1945–1957. 24817139 10.1093/brain/awu100

[bibr10-2398212817701448] Coupe P Manjon JV Gedamu E (2010) Robust Rician noise estimation for MR images. Medical Image Analysis 14(4):483–493. 20417148 10.1016/j.media.2010.03.001

[bibr11-2398212817701448] Das SR Avants BB Pluta J (2012) Measuring longitudinal change in the hippocampal formation from in vivo high-resolution T2-weighted MRI. NeuroImage 60(2): 1266–1279. 22306801 10.1016/j.neuroimage.2012.01.098 PMC3667607

[bibr12-2398212817701448] Dice LR (1945) Measures of the amount of ecologic association between species. Ecology 26(3): 297–302.

[bibr13-2398212817701448] Ding SL Van Hoesen GW (2015) Organization and detailed parcellation of human hippocampal head and body regions based on a combined analysis of cyto- and chemoarchitecture. Journal of Comparative Neurology 523(15): 2233–2253. 25872498 10.1002/cne.23786

[bibr14-2398212817701448] Duvernoy HM Cattin F Risold PY (2013) The Human Hippocampus (4th edn). Berlin; Heidelberg: Springer.

[bibr15-2398212817701448] Green RC Mesulam MM (1988) Acetylcholinesterase fiber staining in the human hippocampus and parahippocampal gyrus. Journal of Comparative Neurology 273(4): 488–499. 3209735 10.1002/cne.902730405

[bibr16-2398212817701448] Iglesias JE Augustinack JC Nguyen K (2015) A computational atlas of the hippocampal formation using ex vivo, ultra-high resolution MRI: Application to adaptive segmentation of in vivo MRI. NeuroImage 115: 117–137. 25936807 10.1016/j.neuroimage.2015.04.042 PMC4461537

[bibr17-2398212817701448] Insausti R Munoz M (2001) Cortical projections of the non-entorhinal hippocampal formation in the cynomolgus monkey ( Macaca fascicularis). European Journal of Neuroscience 14(3): 435–451. 10.1046/j.0953-816x.2001.01662.x 11553294

[bibr18-2398212817701448] Kulaga-Yoskovitz J Bernhardt BC Hong SJ (2015) Multi-contrast submillimetric 3 Tesla hippocampal subfield segmentation protocol and dataset. Scientific Data 2: 150059. 26594378 10.1038/sdata.2015.59 PMC4640139

[bibr19-2398212817701448] La Joie R Perrotin A de La Sayette V (2013) Hippocampal subfield volumetry in mild cognitive impairment, Alzheimer’s disease and semantic dementia. NeuroImage: Clinical 3: 155–162. 24179859 10.1016/j.nicl.2013.08.007 PMC3791274

[bibr20-2398212817701448] Maass A Berron D Libby LA (2015) Functional subregions of the human entorhinal cortex. Elife 4. 10.7554/eLife.06426 PMC4458841 26052749

[bibr21-2398212817701448] Maass A Schutze H Speck O (2014) Laminar activity in the hippocampus and entorhinal cortex related to novelty and episodic encoding. Nature Communications 5: 5547. 10.1038/ncomms6547 PMC4263140 25424131

[bibr22-2398212817701448] Maguire EA Mullally SL (2013) The hippocampus: A manifesto for change. Journal of Experimental Psychology: General 142(4): 1180–1189. 23855494 10.1037/a0033650 PMC3906798

[bibr23-2398212817701448] Mai JK Paxinos G Voss T (2008) Atlas of the Human Brain. New York: Academic Press.

[bibr24-2398212817701448] Malykhin NV Lebel RM Coupland NJ (2010) In vivo quantification of hippocampal subfields using 4.7 T fast spin echo imaging. NeuroImage 49(2): 1224–1230. 19786104 10.1016/j.neuroimage.2009.09.042

[bibr25-2398212817701448] McLardy T (1963) Some cell and fibre peculiarities of uncal hippocampus. Progress in Brain Research 3: 71–88.

[bibr26-2398212817701448] Mueller SG Stables L Du AT (2007) Measurement of hippocampal subfields and age-related changes with high resolution MRI at 4T. Neurobiology of Aging 28(5): 719–726. 16713659 10.1016/j.neurobiolaging.2006.03.007 PMC1820772

[bibr27-2398212817701448] Mugler JP III Bao S Mulkern RV (2000) Optimized single-slab three-dimensional spin-echo MR imaging of the brain. Radiology 216(3): 891–899. 10966728 10.1148/radiology.216.3.r00au46891

[bibr28-2398212817701448] Olsen RK Palombo DJ Rabin JS (2013) Volumetric analysis of medial temporal lobe subregions in developmental amnesia using high-resolution magnetic resonance imaging. Hippocampus 23(10): 855–860. 23749334 10.1002/hipo.22153 PMC4165307

[bibr29-2398212817701448] Palombo DJ Amaral RS Olsen RK (2013) KIBRA polymorphism is associated with individual differences in hippocampal subregions: Evidence from anatomical segmentation using high-resolution MRI. Journal of Neuroscience 33(32): 13088–13093. 23926262 10.1523/JNEUROSCI.1406-13.2013 PMC6619733

[bibr30-2398212817701448] Poppenk J Evensmoen HR Moscovitch M (2013) Long-axis specialization of the human hippocampus. Trends in Cognitive Sciences 17(5): 230–240. 23597720 10.1016/j.tics.2013.03.005

[bibr31-2398212817701448] Rosene DL van Hoesen GW (1987) The hippocampal formation of the primate brain. In: Jones EG Peters A (eds) Cerebral Cortex (Vol. 6). New York: Springer, pp. 345–456.

[bibr32-2398212817701448] Sasaki M Sone M Ehara S (1993) Hippocampal sulcus remnant: Potential cause of change in signal intensity in the hippocampus. Radiology 188(3): 743–746. 8351342 10.1148/radiology.188.3.8351342

[bibr33-2398212817701448] Satpute AB Mumford JA Naliboff BD (2012) Human anterior and posterior hippocampus respond distinctly to state and trait anxiety. Emotion 12(1): 58–68. 22309734 10.1037/a0026517

[bibr34-2398212817701448] Schacter DL Addis DR Hassabis D (2012) The future of memory: Remembering, imagining, and the brain. Neuron 76(4): 677–694. 23177955 10.1016/j.neuron.2012.11.001 PMC3815616

[bibr35-2398212817701448] Strange BA Witter MP Lein ES (2014) Functional organization of the hippocampal longitudinal axis. Nature Reviews Neuroscience 15(10): 655–669. 25234264 10.1038/nrn3785

[bibr36-2398212817701448] Van Leemput K Bakkour A Benner T (2009) Automated segmentation of hippocampal subfields from ultra-high resolution in vivo MRI. Hippocampus 19(6): 549–557. 19405131 10.1002/hipo.20615 PMC2739884

[bibr37-2398212817701448] Van Veluw SJ Wisse LE Kuijf HJ (2013) Hippocampal T2 hyperintensities on 7 Tesla MRI. NeuroImage: Clinical 3: 196–201. 24179863 10.1016/j.nicl.2013.08.003 PMC3791290

[bibr38-2398212817701448] Winterburn JL Pruessner JC Chavez S (2013) A novel in vivo atlas of human hippocampal subfields using high-resolution 3 T magnetic resonance imaging. NeuroImage 74: 254–265. 23415948 10.1016/j.neuroimage.2013.02.003

[bibr39-2398212817701448] Winterburn JL Pruessner JC Sofia C (2015) High-resolution in vivo manual segmentation protocol for human hippocampal subfields using 3T magnetic resonance imaging. Journal of Visualized Experiments 105: e51861. 10.3791/51861 PMC4692694 26575133

[bibr40-2398212817701448] Wisse LE Gerritsen L Zwanenburg JJ (2012) Subfields of the hippocampal formation at 7 T MRI: In vivo volumetric assessment. NeuroImage 61(4): 1043–1049. 22440643 10.1016/j.neuroimage.2012.03.023

[bibr41-2398212817701448] Wood B Knight MJ Tsivos D (2015) Magnetic resonance scanning and image segmentation procedure at 3 T for volumetry of human hippocampal subfields. Biomedical Spectroscopy and Imaging 4: 197–208.

[bibr42-2398212817701448] Yushkevich PA Amaral RS Augustinack JC (2015) Quantitative comparison of 21 protocols for labeling hippocampal subfields and parahippocampal subregions in in vivo MRI: Towards a harmonized segmentation protocol. NeuroImage 111: 526–541. 25596463 10.1016/j.neuroimage.2015.01.004 PMC4387011

[bibr43-2398212817701448] Yushkevich PA Avants BB Pluta J (2009) A high-resolution computational atlas of the human hippocampus from postmortem magnetic resonance imaging at 9.4 T. NeuroImage 44(2): 385–398. 18840532 10.1016/j.neuroimage.2008.08.04 PMC2650508

[bibr44-2398212817701448] Yushkevich PA Piven J Hazlett HC (2006) User-guided 3D active contour segmentation of anatomical structures: Significantly improved efficiency and reliability. NeuroImage 31(3): 1116–1128. 16545965 10.1016/j.neuroimage.2006.01.015

[bibr45-2398212817701448] Zeidman P Maguire EA (2016) Anterior hippocampus: The anatomy of perception, imagination and episodic memory. Nature Reviews Neuroscience 17(3): 173–182. 26865022 10.1038/nrn.2015.24 PMC5358751

[bibr46-2398212817701448] Zeidman P Lutti A Maguire EA (2015) Investigating the functions of subregions within anterior hippocampus. Cortex 73: 240–256. 26478961 10.1016/j.cortex.2015.09.002 PMC4686003

